# A probiotic treatment increases the immune response induced by the nasal delivery of spore-adsorbed TTFC

**DOI:** 10.1186/s12934-020-01308-1

**Published:** 2020-02-19

**Authors:** Francisco Denis S. Santos, Arianna Mazzoli, Ana Raquel Maia, Anella Saggese, Rachele Isticato, Fabio Leite, Susanna Iossa, Ezio Ricca, Loredana Baccigalupi

**Affiliations:** 1 Dipartimento di Biologia, Università di Napoli Federico II, Naples, Italy; 2 Centro de Desenvolvimento Tecnológico, Núcleo de Biotecnologia, Universidade Federal de Pelotas, Pelotas, Brazil; 3 Dipartimento di Medicina Molecolare e Biotecnologie Mediche, Università di Napoli Federico II, Naples, Italy

**Keywords:** Mucosal vaccine, Mucosal adjuvant, *Bacillus*, Gut, 16S analysis

## Abstract

**Background:**

Spore-forming bacteria of the *Bacillus* genus are widely used probiotics known to exert their beneficial effects also through the stimulation of the host immune response. The oral delivery of *B. toyonensis* spores has been shown to improve the immune response to a parenterally administered viral antigen in mice, suggesting that probiotics may increase the efficiency of systemic vaccines. We used the C fragment of the tetanus toxin (TTFC) as a model antigen to evaluate whether a treatment with *B. toyonensis* spores affected the immune response to a mucosal antigen.

**Results:**

Purified TTFC was given to mice by the nasal route either as a free protein or adsorbed to *B. subtilis* spores, a mucosal vaccine delivery system proved effective with several antigens, including TTFC. Spore adsorption was extremely efficient and TTFC was shown to be exposed on the spore surface. Spore-adsorbed TTFC was more efficient than the free antigen in inducing an immune response and the probiotic treatment improved the response, increasing the production of TTFC-specific secretory immunoglobin A (sIgA) and causing a faster production of serum IgG. The analysis of the induced cytokines indicated that also the cellular immune response was increased by the probiotic treatment. A 16S RNA-based analysis of the gut microbial composition did not show dramatic differences due to the probiotic treatment. However, the abundance of members of the *Ruminiclostridium* 6 genus was found to correlate with the increased immune response of animals immunized with the spore-adsorbed antigen and treated with the probiotic.

**Conclusion:**

Our results indicate that *B. toyonensis* spores significantly contribute to the humoral and cellular responses elicited by a mucosal immunization with spore-adsorbed TTFC, pointing to the probiotic treatment as an alternative to the use of adjuvants for mucosal vaccinations.

## Introduction

Mucosal surfaces are the most common route used by pathogens to enter the human and animal body. For this reason, it is extremely important for a vaccine to induce secretory immunoglobin A (sIgA) antibody production and elicit immune protection at the mucosal surfaces [1]. While injected vaccines induce specific T cell responses in the bloodstream and serum IgG production but generally fail to induce sIgA, mucosal vaccines administered via the oral or nasal routes induce humoral and cellular immune responses at both the systemic and mucosal sites [2, 3]. Therefore, mucosal, needle-free vaccines are potentially preferable over parenteral vaccinations [4]. However, only few mucosal vaccines are currently licensed for vaccination against viral (Rotavirus, Poliovirus, Influenza type A virus) or bacterial (*Salmonella typhi, Vibrio cholerae*) pathogens [3]. This is mostly due to the low immunogenicity of most mucosal antigens and to the lack of efficient adjuvants and delivery systems [4]. Indeed, adjuvants commonly used in injected vaccines fail to induce sIgA and therefore are not efficient with mucosal antigens, while the lack of appropriate delivery systems does not prevent antigen degradation by enzymes present in the mucosal tissues [3].

Major efforts have been devoted to the development of new mucosal vaccination strategies based on adjuvants able to induce sIgA or on novel delivery systems based on synthetic nanoparticles, viral particles, microbial cells or bacterial spores [5–8].

The use of probiotics before and/or during the vaccination period to modulate the immune response [9] and increase the effectiveness of vaccines against bacterial [9, 10] or viral [11, 12] infections is also receiving increasing interest. In a recent study, spores of *Bacillus toyonensis* were shown able to increase the immune response to a parenteral vaccine against bovine herpesvirus type 5 (BoHV-5) in mice [13]. *B. toyonensis,* originally defined as *B. cereus* var. toyoi and then identified as a new species by genomic analysis [14], was used in animal nutrition for swine, poultry, cattle, rabbits and aquaculture. In 1994 its use has been authorized by the European Community as a feed additive for use in poultry, cattle and rabbits [15]. Animals parenterally immunized with BoHV-5 and orally supplemented with *B. toyonensis* spores had higher serum IgG, IL-4 and IL-12 levels than immunized animals that did not receive the probiotic [13], suggesting this probiotic treatment as a potential alternative to the use of adjuvants.

The aim of this work was to investigate whether the oral treatment with spores of *B. toyonensis* was also effective in inducing the production of specific sIgA thus improving the immune response induced by a mucosal antigen. The C fragment of the tetanus toxin (TTFC), the protective antigen used in evaluations of vaccines against tetanus, was selected as a model antigen [16]. TTFC administered by the oral or nasal route was shown to induce a protective immune response in mice when delivered by *B. subtilis* spores either as a fusion protein exposed on the spore surface [17–19] or as a pure protein adsorbed on the spore surface [20].

The use of *B. subtilis* spores as a mucosal delivery system has been exploited in recent years and tested with several antigens and enzymes [6, 21, 22]. In addition to TTFC, the binding subunit of the heat-labile toxin (LTB) of *Escherichia coli* [23, 24], the protective antigen (PA) of *B. anthracis* [20], the C terminus of toxin A of *Clostridium difficile* [25], the capsid proteins VP26 and VP28 of the White Spot Syndrome virus [26, 27] and the MPT64 antigen of *Mycobacterium tuberculosis* [28] are examples of antigens displayed by *B. subtilis* spores and tested as mucosal vaccines.

## Results and discussion

### Spore adsorption of the C fragment of the tetanus toxin (TTFC)

**Fig. 1:**
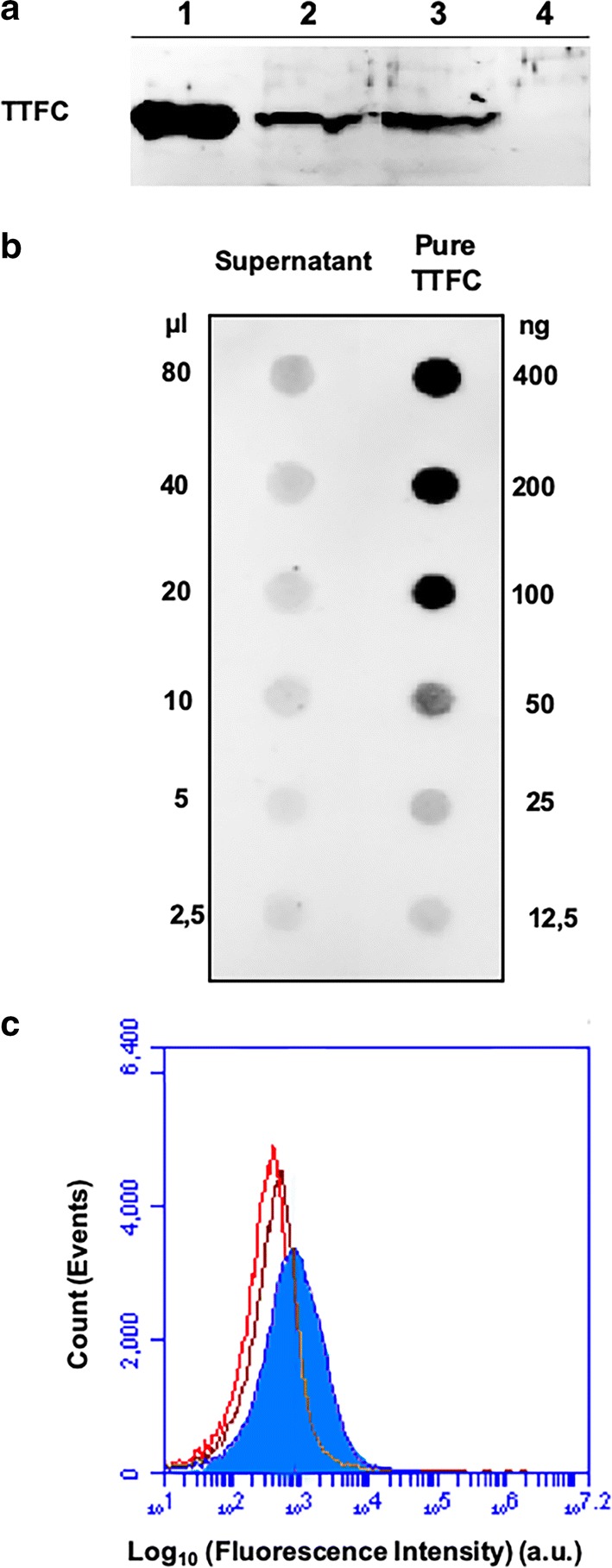
TTFC adsorption on *B. subtilis* spores. **a** Western blotting of spore surface proteins after adsorption with 2.0 µg of purified TTFC. Lanes 1: purified TTFC; 2: proteins extracted from adsorbed spores; 3: proteins extracted from adsorbed spores after 1 week storage at 4 °C; 4: five-fold concentrated supernatant after 1 week storage at 4 °C. **b** Dot blotting experiment performed with the serial dilutions of the supernatant (unbound TTFC) fraction of the adsorption reaction. Serial dilutions of purified TTFC were used as a standard. **c** Flow  cytometry analysis of: free spores incubated (brown histogram) or not (red histogram) with specific antibodies and TTFC-adsorbed spores incubated with specific antibodies (filled blue histogram). The analysis was performed on the entire spore population (ungated). Immune-reactions were performed with polyclonal anti-TTFC [17] and anti-rabbit HRP conjugate (panels A and B) or with FITC-conjugated secondary antibodies (panel C)

Aliquots (2.0 μg) of TTFC, over-expressed in *E. coli* and purified by affinity chromatography columns (Methods), were incubated in 200 μl of 50 mM sodium citrate buffer at pH 4.0 with 2.0 × 10^9^ spores of the *B. subtilis* strain PY79 [29], purified as previously described [30]. After 1 h of incubation at 25 °C spores were collected by centrifugation and surface proteins extracted by SDS-DTT treatment [31]. Proteins were then analyzed by western blotting with anti-TTFC antibody [17] and TTFC was found among the proteins extracted from the spore surface (Fig. 1a), as previously reported [20]. To assess the stability of spore-TTFC interaction, spores adsorbed with TTFC were re-suspended in 200 μl of 50 mM sodium citrate buffer at pH 4.0 and stored 1 week at 4 °C. Upon centrifugation, spores were used to extract surface proteins as described above while the supernatant was five-fold concentrated by ultra-filtration (3 kDa cut-off) and analyzed by western blotting. As shown in Fig. 1a, TTFC was still extracted from 1-week-old spores (lane 3) and was not present in the supernatant (lane 4), indicating that TTFC was not degraded and or released during the storage at 4 °C.

To indirectly quantify the amount of TTFC adsorbed on the spore, the adsorption reaction mixture was fractionated by centrifugation and the supernatant, containing the unbound, free TTFC was analyzed by dot blotting with anti-TTFC antibody (Fig. 1b). The intensity of the various spots was then quantified by a densitometry analysis as previously described [22] and indicated that in our experimental conditions less than 3% of TTFC was left free in the supernatant (Table 1). Such a high efficiency of adsorption was not surprising since previous reports have shown that in similar experimental conditions over 90% of reacted proteins were adsorbed to *B. subtilis* spores [22, 24].

**Table 1:** Densitometric analysis of dot blot experiments of Fig. 1b with the supernatants of the adsorption reaction with wild type spores

TTFC source	Amount of sample used	Density (OD/mm^2^)	Amount of TTFC (ng)	Amount of TTFC μg in 200 μl (% total)
Purified TTFC	50.00 ng	29.053	NA	NA
	25.00 ng	13.121	NA	NA
	12.50 ng	5.294	NA	NA
Free TTFC	80 μl	16.505	23.4	0.05 (2.9)
(supernatant)	40 μl	8.012	11.8	0.05 (2.9)
	20 μl	4.629	4.5	0.04 (2.2)

A flow cytometry approach was used to evaluate the exposure of TTFC on the spore surface. Spores adsorbed with TTFC were reacted with anti-TTFC specific antibody, then with FITC-conjugated secondary antibody and analyzed by flow cytometry (Fig. 1c). In parallel, free spores incubated or not with antibodies (primary and secondary) were analyzed to take into consideration the unspecific fluorescence of spores (Fig. 1c, brown and red histogram, respectively). These controls, overlaid and used as a reference guide in the measurement of the TTFC-specific fluorescence, indicated that when adsorbed with TTFC the majority of the spore population (64% of the 100,000 counted spores) were specifically fluorescent and, therefore, displayed the antigen (Additional file 1: Figure S1).

### A probiotic treatment increases sIgA production induced by a nasal administration of spore-adsorbed TTFC

**Fig. 2:**
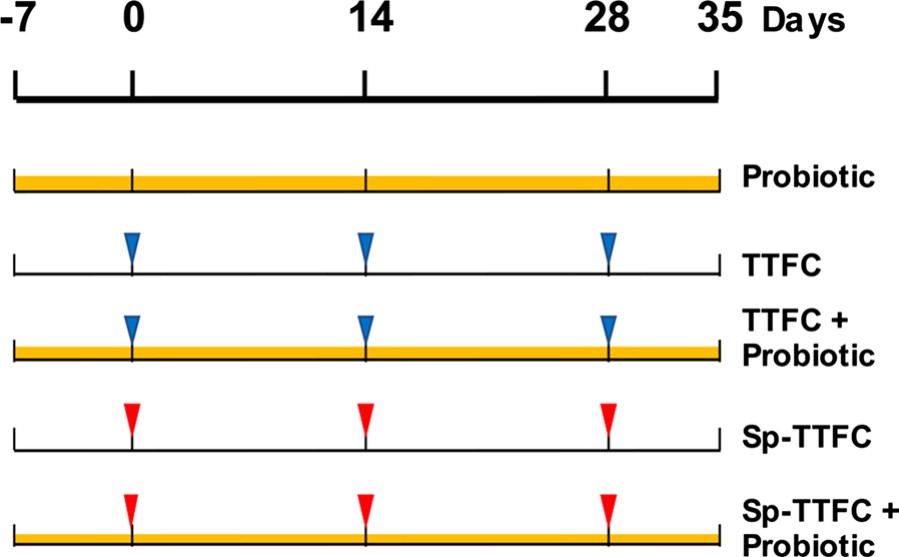
Experimental plan. Three experimental groups received the oral probiotic treatment (yellow lines) with 1.0 × 10^6^ spores/gram from day -7 to day 35. One of these groups was immunized with 2.0 μg of purified TTFC (blue arrows) and another one with 2.0 × 10^9^ spores adsorbed with TTFC (Sp-TTFC) (red arrows) on day 0, 14 and 28. All immunizations were performed by the nasal route. Two groups were immunized only with purified TTFC or Sp-TTFC without probiotics. A naive group that did not receive either probiotics or the antigen was also included. Blood samples were collected from all animals at days 0, 14 and 21 and 35, at day 35 all animals were sacrificed for analysis

In a previous study [20], spore-adsorbed TTFC was administered by the nasal route to mice and shown able to induce an antigen-specific mucosal response. We used the same dosage and administration route used before [20] to assess whether a probiotic treatment with *B. toyonensis* was able to influence the mucosal immune response elicited by spore-adsorbed TTFC. To evaluate the effect of the probiotic on the immune response induced by the pure antigen, parallel groups of animals were also immunized with 2.0 μg of purified TTFC. Figure 2 schematically shows the experimental plan: three groups of animals received the oral probiotic treatment (1.0 × 10^6^ spores/gram of food from day -7 to day 35), two groups were immunized with 2.0 μg of purified TTFC by the nasal route on day 0, 14 and 28 (blue arrows in Fig. 2) and two groups received 2.0 × 10^9^ spores adsorbed with TTFC by the nasal route on day 0, 14 and 28 (red arrows in Fig. 2). A naive group that did not receive either probiotics or the antigen was also included. Blood samples were collected from all animals at days 14 and 21 and at day 35 all animals were sacrificed for analysis. As calculated in the previous paragraph, 2.0 × 10^9^ spores adsorbed with 2.0 μg of TTFC displayed about 1.9 μg of TTFC (over 90% of the total TTFC), therefore, three doses of spores delivered a total 5.7 μg of TTFC, slightly less than the amount of antigen received by the animals immunized with the purified antigen (6 μg).

**Fig. 3:**
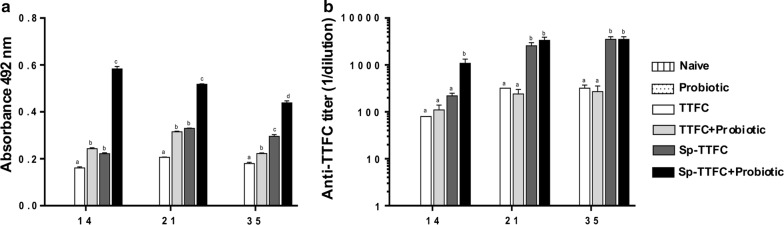
Antibody production. **a** Anti-TTFC specific fecal sIgA detected on days 14, 21 and 35. Data were expressed as the mean (± standard error) of absorbance values at 492 nm. **b** Anti-TTFC specific serum IgG detected on days 14, 21, and 35. Not-immunized (naïve and probiotic) groups did not produce anti-TTFC antibodies and were not reported in the figure. The data represent the mean (± standard error) of reciprocal endpoint titers. Equal letters mean no statistical difference (p > 0.05) and different letters mean a statistical difference (p < 0.05) between the experimental groups

High anti-TTFC fecal sIgA levels, indicative of a mucosal immune response, were induced by spore-adsorbed TTFC in animals treated with the probiotic (Fig. 3a). The response was maximal after 14 days and slightly decreased at days 21 and 35. As expected, the free antigen did not induce high levels of sIgA and the treatment with the probiotic caused only a minimal increase (Fig. 3a). The analysis of serum antibodies showed a positive effect of the probiotic on the immune response induced by spore-adsorbed TTFC at day 14 (Fig. 3b). At days 21 and 35 similar levels of IgG were induced by spore-adsorbed TTFC with or without the probiotic treatment (Fig. 3b). Low levels of TTFC-specific IgG were induced by the purified antigen after 14 days, those levels were slightly increased after 21 and 35 days and were not affected by the treatment with the probiotic (white and light grey bars in Fig. 3b, respectively). The ability of nasally administered spore-adsorbed TTFC to induce a stronger immune response than purified TTFC, at days 21 and 35 (compare white and dark grey bars in Fig. 3b), could be due to an increased antigen uptake by immune cells or, alternatively, to a reduced antigen degradation, as previously suggested for another antigen [24]. Additional experiments are required to fully address this issue. For the aim of this work, it is noteworthy that the probiotic increased the mucosal (sIgA) immune response and accelerated the production of serum IgG induced to spore-adsorbed TTFC.

**Fig. 4:**
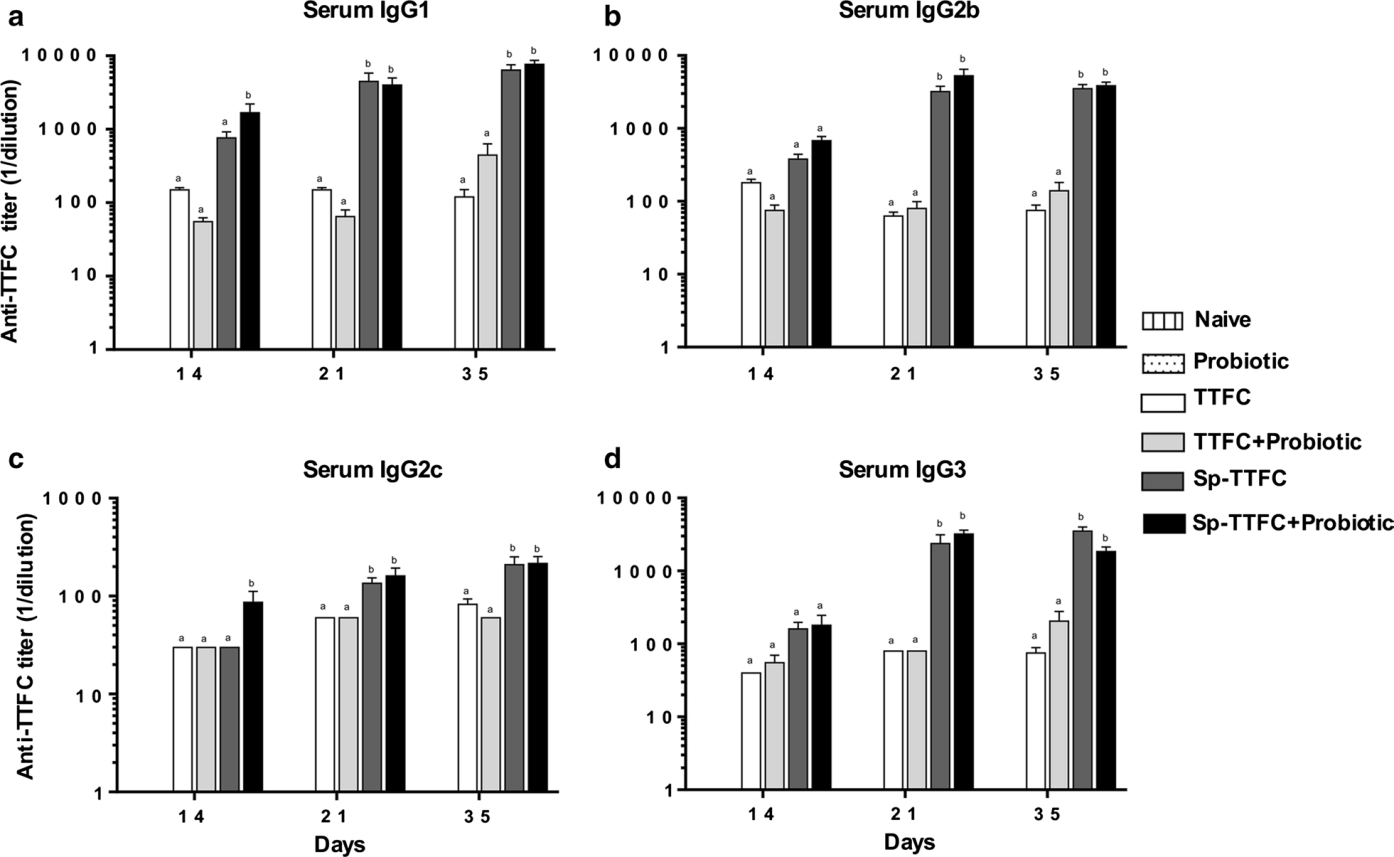
IgG subclasses analysis. The phenotype of the induced humoral immune response. Anti-TTFC IgG1 (**a**), IgG2b (**b**), IgG2c (**c**), and IgG3 (**d**) levels detected in mice serum on days 14, 21, and 35. Not-immunized (naïve and probiotic) groups did not produce anti-TTFC antibodies and were not reported in the figure. The data represent the mean (± standard error) of reciprocal endpoint titers. Equal letters mean no statistical difference (p > 0.05) and different letters mean a statistical difference (p < 0.05) between the experimental groups

The phenotype of the induced humoral immune response was then examined analyzing IgG subclasses. High levels of IgG1, IgG2b, IgG2c or IgG3 subtypes were induced at all time-points in animals immunized with Sp-TTFC, independently from the probiotic treatment (Fig. 4). Only at day 14 was IgG2c higher in probiotic-treated animals than in those that did not receive *B. toyonensis* (Fig. 4b). Since in mice, the IgG1 isotype is associated with a Th2 response, whereas IgG2c (analogous to IgG2a in other mouse strains) and IgG2b sometimes associated with IgG3 reflect a Th1 response [32, 33], results of Fig. 4 suggest the induction of potent and mixed Th1/Th2-type immune responses elicited by spore-adsorbed TTFC independently of the probiotic treatment.

Altogether, the results of Fig. 3, 4, indicate that the treatment with *B. toyonensis* spores increases fecal sIgA production in animals nasally immunized with TTFC carried by *B. subtilis* spores while it does not affect the level and the phenotype of the serum IgG response.

### A probiotic treatment increases the cellular immune response elicited by a nasal administration of spore-adsorbed TTFC

**Fig. 5:**
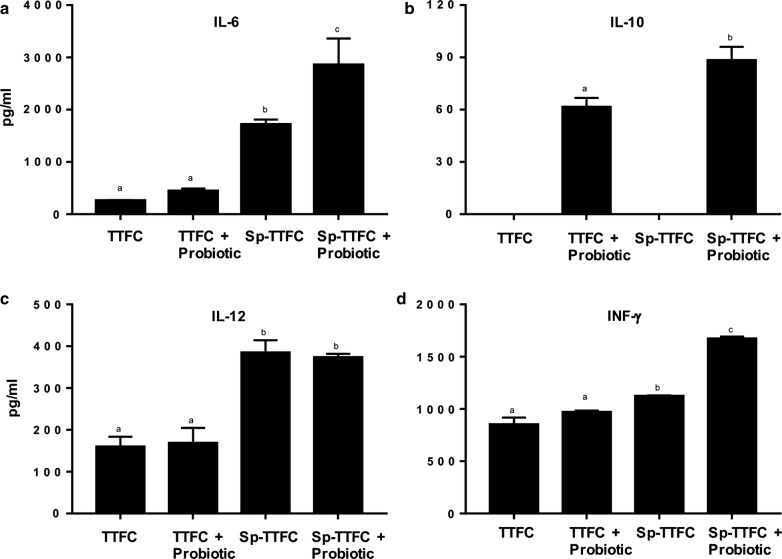
Cytokine induction. The cellular immune response elicited by TTFC and spore-adsorbed TTFC treated with probiotic. IL-6 (**a**), IL-10 (**b**), IL-12 (**c**), and IFN-γ (**d**) levels secreted in vitro from spleen cells. The results were expressed as pg/ml of the mean values (± standard error). Data are reported after subtracting the cytokine values detected in control groups (naive and not immunized mice that received the probiotic). Equal letters mean no statistical difference (p > 0.05) and different letters mean a statistical difference (p < 0.05) between the experimental groups

The spleen of all vaccinated animals was analyzed for TTFC-specific production of cytokines IL-4, IL-6, IL-10, IL-12 and IFN-γ. While IL-4 was not produced at detectable levels (not shown), all other analyzed cytokines were detected in the culture supernatants. High levels of IL-6 were produced by splenocytes from mice that received spore-adsorbed TTFC treated and not treated with the probiotic, however, in probiotic-treated animals IL-6 levels were statistically higher (Fig. 5a). The IL-6 is a pro-inflammatory cytokine that plays a central role during the transition from innate to adaptive immunity [34]. Recent studies showed that IL-6 induces the maturation of B cells into antibody-secreting cells and promotes the survival and maintenance of long-lived plasma cells [35].

IL-10 was detected only in the spleen of mice immunized with either pure TTFC or spore-bound TTFC that were treated with the probiotic (Fig. 5b). Animals treated with the probiotic but not immunized only showed basal levels of IL-10. Results on IL-10 are consistent with recent reports showing an increase in IL-10 expression in splenocytes of animals supplemented with *B. toyonensis* spores and vaccinated with a parenteral vaccine against bovine herpesvirus type 5 [12, 36]. IL-10 is a cytokine that can be produced by a number of cell types including T cells, B cells and macrophages and acts controlling the intensity of the immune response [37], increasing the survival of B cells, increasing the production of immunoglobulins, and mediating the immune stimulatory effects on T cells [38].

The probiotic treatment did not affect the production of IL-12 that was low in the spleen of mice immunized with TTFC and high in mice immunized with Sp-TTFC, independently from the probiotic treatment (Fig. 5c). Instead, *B. toyonensis* spores were able to increase the IFN-γ levels produced by spleen cells of mice vaccinated with Sp-TTFC (Fig. 5d). IFN-γ directs the differentiation of naïve T lymphocytes into Th1 cells [39], and the induction of a Th1 type of immune response by spores is in agreement with previous reports on spores displaying antigens [20, 24].

Overall, the results of Fig. 5 indicate that the probiotic treatment increases the cellular response to nasally administered TTFC carried by *B. subtilis* spores.

### The probiotic treatment did not strongly alter the microbial composition of the animal gut

A 16S DNA-sequencing approach was used to investigate the effect of the probiotic treatment on the gut microbial composition. As reported below, the analysis performed on samples of animals of the control group was in agreement with previous data for mice, with Firmicutes much more abundant than Bacteroidetes [40].

**Fig. 6:**
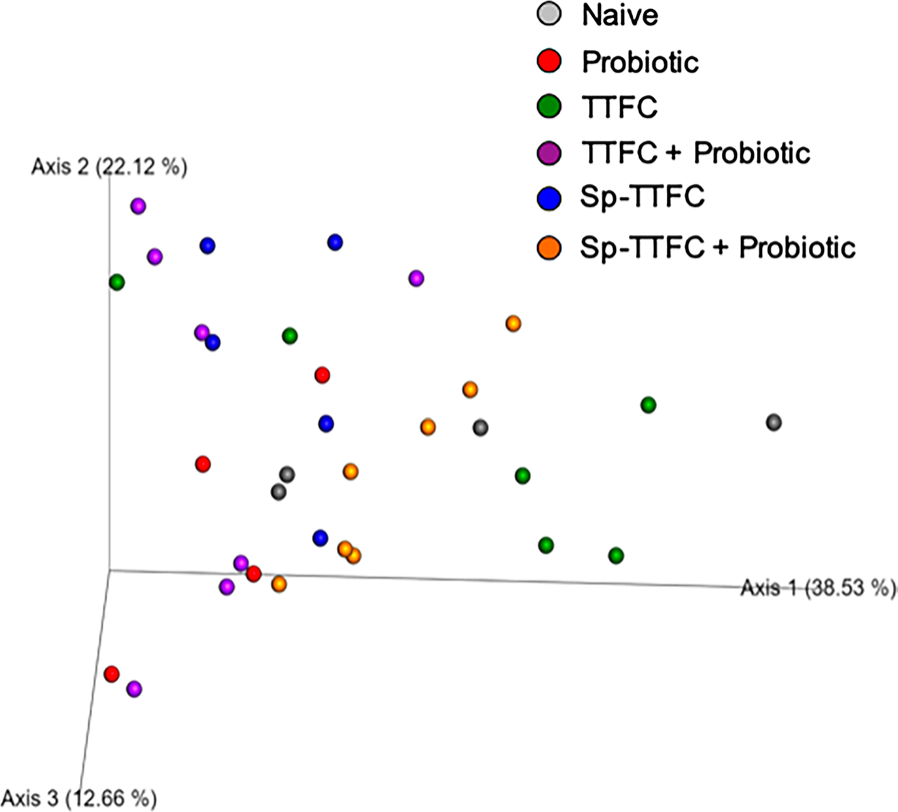
Principal Coordinate Analysis (PCoA). Plots were generated using weighted UniFrac distance matrix

**Fig. 7:**
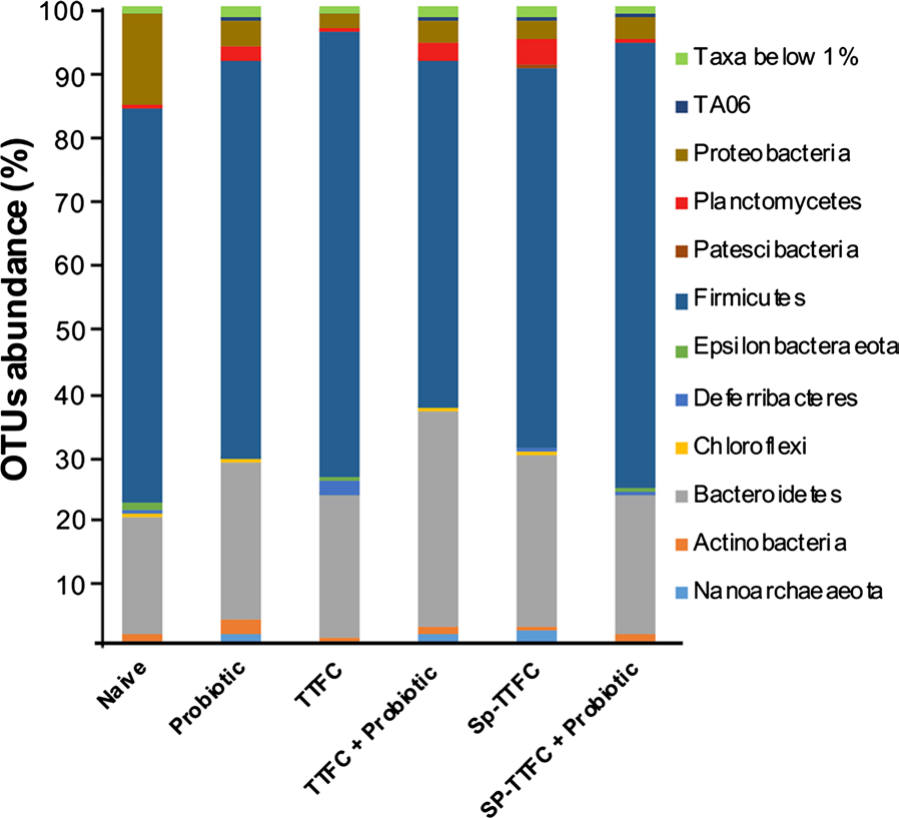
Fecal bacterial composition. Relative Operational Taxonomic Units (OTUs) abundance at the Phylum level in the six experimental groups, reported as mean values within each group. Only Taxa represented by OTUs abundance > 1% have been considered for the analysis

**Fig. 8:**
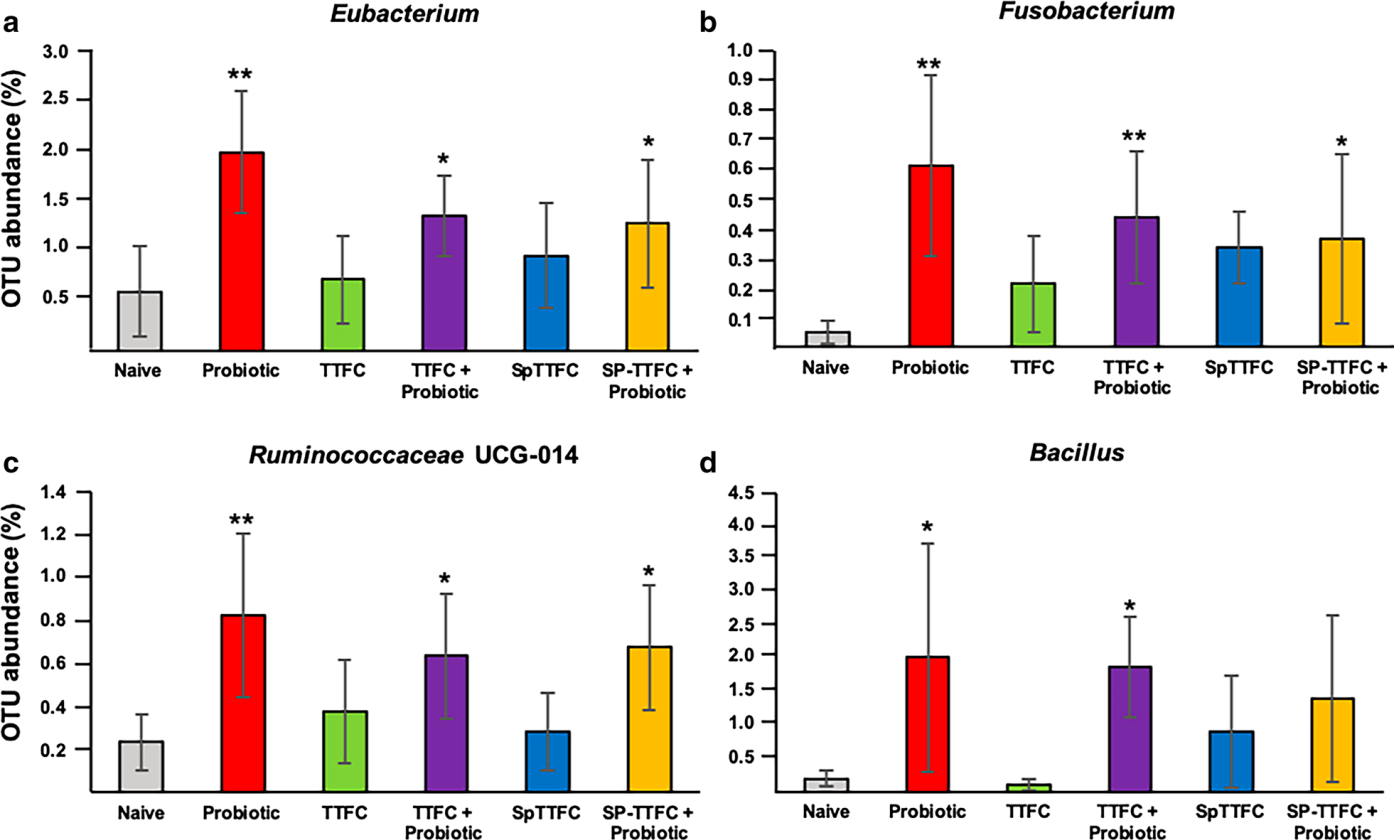
Representativeness of four bacterial genera. The different abundance of four genera between the probiotic treated groups and the control is reported. Statistically significant differences are indicated by asterisks (* = p<0.05; ** = p<0.005)

PCoA based on Bray–Curtis distance showed that the gut microbiota of mice of the various groups did not form clear separate clusters, suggesting that the immunizations and/or probiotic treatments did not dramatically alter the microbial composition of the animal gut (Fig. 6). The OTU representation curves indicated that the microbial diversity of the samples was completely covered while the alpha-diversity analysis showed a higher number of species in two animals of the control (naive) group than in all other groups that did not differ significantly among each other (Additional file 2: Figure S2). The analysis of the bacterial composition, reported as the average of the relative abundance of bacterial taxa at the phylum, family and genus level, did not show dramatic differences among the experimental groups. The identified phylotypes showed that Firmicutes were the most abundant bacteria in all groups (54–70%) while Bacteroidetes and Proteobacteria were always less represented (18–33% and 2–14%, respectively) with the latter Phylum that was less represented in all experimental groups with respect to the naive group (Fig. 7). The analysis at the family and genus level (Additional file 3: Figure S3) was, then, focused on the bacterial taxa of the various groups that showed a statistically significant variation (p < 0.05) in their representation with respect to the naive group. By this approach three bacterial genera were found to have a statistically different representation between the probiotic-supplemented and naive groups: *Eubacterium* (Fig. 8a), *Fusobacterium* (Fig. 8b) and *Ruminococcaceae* UCG-014 (Fig. 8c). In addition, the *Bacillus* genus which includes species used here as the probiotic and the antigen delivery vehicle, was differently represented between probiotic-supplemented and naive groups (Fig. 8d). However, in this case the difference was statistically significant only for two of the three groups (Fig. 8d). Altogether, the results of Fig. 8 indicate that the probiotic treatment did not drastically affect the gut microbial composition but instead altered the abundance of few genera.

**Fig. 9:**
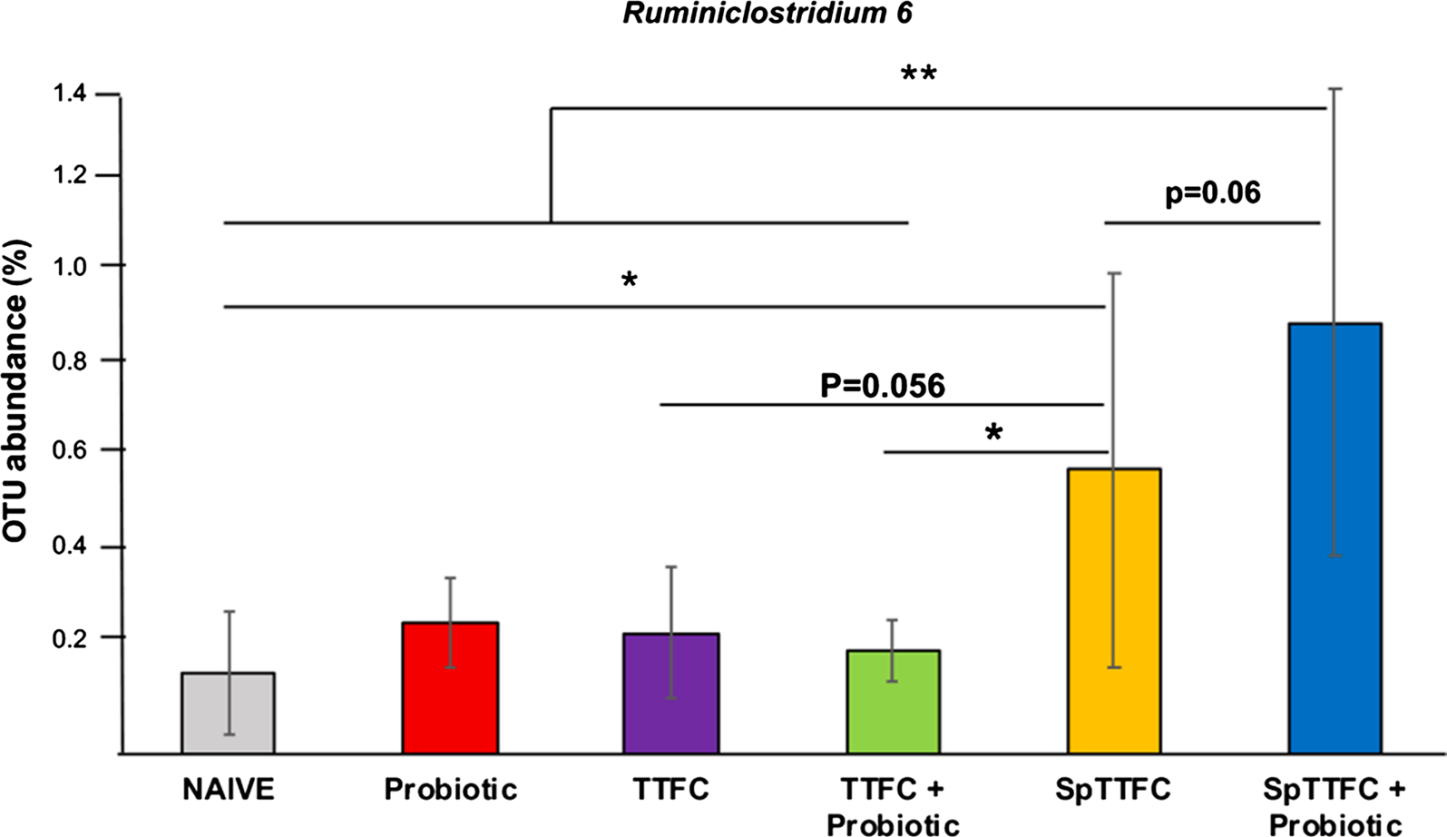
Representativeness of the *Ruminiclostridium* 6 genus. The different abundance of *Ruminiclostridium* 6 between groups immunized with Sp-TTFC and the other groups is reported. Statistically significant differences are indicated by asterisks (* = p<0.05; ** = p<0.005). Differences with p value slightly above the threshold are also shown

Additionally we analysed the statistically relevant differences between genera in the two groups that gave better immune responses (Sp-TTFC and Sp-TTFC+Probiotic) respect to all other groups. By this approach we found that members of *Ruminiclostridium* 6 genus were abundant in the gut of animals immunized with spore-displayed TTFC that received the probiotic (Fig. 9). The same genus was also abundant in the gut of animals of the Sp-TTFC group, however, the differences were statistically significant with the naive,, and TTFC+probiotic groups, slightly above the threshold (p < 0.05) with the TTFC group and not statistically significant with the probiotic group (Fig. 9).

The correlation between the abundance of *Ruminiclostridium* 6 and a high immune response was analyzed by the Pearson method. As reported in Table 2, *Ruminiclostridium* abundance nicely correlated with the fecal IgA (ρ = 0.892 with p < 0.05) and serum IgG (ρ = 0.937 with p < 0.05). A positive correlation was also found with IL-6 (ρ = 0.995 with p < 0.05) accordingly with recently reported data [41]. In the case of the other cytokines analyzed in this study, the correlation was not statistically significant (p > 0.05) (Table 2).

**Table 2:** Correlation analysis between *Ruminiclostridium* 6 abundance and immune response

	Correlation index (ρ)^a^	p value
Fecal IgA	0.892	0.0416
Serum IgG	0.937	0.0190
IL-6	0.995	0.000368
IL-12	0.876	0.0512
IFN-g	0.812	0.0947
IL-10	0.406	0.498

^a^ Performed according to the Pearson method [52]

## Conclusions

The main conclusion of this manuscript is that a probiotic treatment with *B. toyonensis* spores positively affects a nasal immunization with the C fragment of the tetanus toxin (TTFC) displayed by *B. subtilis* spores. While it was already known that *B. toyonensis* spores increased the immune response to a systemic vaccination [13], their efficacy as adjuvant of a mucosal vaccination was never tested before. The observed increased production of fecal sIgA and of IL-6, IL-10 and IFN-γ in the spleen of immunized animals in response to the probiotic treatment clearly points to the *B. toyonensis* spore as a potential mucosal adjuvant.

*B. toyonensis* spores also increased the serum IgG production in animals immunized with spore-adsorbed TTFC. However, this effect was only observed at early, day 14, and not at late, day 21 or 35, time points suggesting that the probiotic cause a faster serum IgG response, probably driven by the IgG2c subclass.

The analysis of the gut microbiota did not show dramatic changes in the various experimental groups. Three genera, *Eubacterium, Fusobacterium* and *Ruminococcaceae* UCG-014, were found to have statistically significant differences in their representation between the naive group and the groups that received the probiotic treatment. Members of the *Eubacterium* genus belong to the *Lachnospiraceae* family and are anaerobic, Gram-positive, non-spore-forming rods, previously associated with dietary fiber-induced modulation of the human gut microbiota [42]. Bacteria of the *Fusobacterium* are obligate anaerobe, Gram-negative rods commonly found as components of the normal flora of the human oropharynx. Some species of the *Fusobacterium* genus are considered as pathogenic, have been associated with colon cancer or found to increase in response to other infections [43]. The *Ruminococcaceae* UCG-014 genus members are obligate anaerobes belonging to the *Ruminococcaceae* family, which contains also other genera commonly found in the animal gut [44]. This analysis then indicates that although the probiotic treatment did not drastically affect the gut microbial composition, it altered the relative abundance of few genera. However, those differences did not correlate with the different immune responses observed.

By comparing the gut microbiota of the two experimental groups that gave better immune responses (Sp-TTFC and Sp-TTFC + Probiotic) vs. all other groups, *Ruminiclostridium* 6 was found statistically more abundant in the Sp-TTFC + Probiotic group. This observation points to a correlation between the abundance of the *Ruminiclostridium* 6 genus and the induction of a strong immune response. Such a positive correlation was demonstrated by the Pearson analysis, showing a statistically significant link between *Ruminiclostridium* 6 abundance and IgG, IgA and IL-6 levels. For two other inflammatory cytokines, IL12 and IFNg, the positive correlation was not supported by the statistical analysis (p > 0.05) even if the p values were slightly above to the threshold. No correlation was found with the anti-inflammatory cytokine IL10.

## Methods

### Bacterial strains, spore and TTFC production

The *B. subtilis* strain PY79 [29] was used in this study and sporulation was induced by the exhaustion method [45]. After 30 h of growth in Difco Sporulation (DS) medium at 37 ºC with vigorous shaking, spores were collected, washed three times with distilled water and purified as described before [30]. Spore counts were determined by serial dilution and plating counting.

The TTFC (tetanus toxin fragment C) from *C. tetani* was expressed from recombinant plasmid (pET-28b) in the *E. coli* strain BL21. The plasmid pET-28b-TTFC expressed *C. tetani* TTFC as a 52.6 kDa polypeptide and has been described elsewhere [17]. The expressed protein carried a poly-histidine tag at its 3′-end and following expression was purified using His-Trap column as recommended by the manufacturer (*GE Healthcare Life Science*).

*B. toyonensis* BCT-7112^T^ used in this study was obtained from the collection of microorganisms of the Microbiology Laboratory, Biotechnology Center, Federal University of Pelotas (Brazil). Bacteria were grown in DS medium at 37ºC for 96 h as previously reported [13] and analyzed under the optical microscope for the presence of cells and spores. The cultures containing over 95% of free spores were centrifuged at 5000*g* for 20 min at 4 °C and the pellet suspended in phosphate buffer to a concentration of spores of approximately 2.0 × 10^7^ CFU/ml.

### Adsorption reaction, western- and dot-blotting analysis

The adsorption reaction was performed by mixing purified TTFC (2.0 µg) and 2.0 × 10^9^ spores in 50 mM Sodium Citrate pH 4.0 at 25 ºC in a final volume of 200 µl. After 1 h of incubation, the binding mixture was centrifuged (10 min at 13,000*g*) to fractionate pellet and supernatant and stored at 4 ºC [31]. The pellet fraction, containing TTFC-adsorbed spores (2.0 × 10^9^) was suspended in 20 μl of spore coat extraction buffer [31], incubated at 68 °C for 1 h to solubilize spore coat proteins and loaded onto a 12% SDS-PAGE gel. The proteins were then electro-transferred to nitrocellulose filters (Amersham Pharmacia Biotech) and used for Western blotting analysis as previously reported [24] using anti-TTFC specific rabbit polyclonal antibodies [17] and Goat Anti-Rabbit (H + L)-HRP Conjugate (Bio-rad). A quantitative determination of the amount of TTFC was obtained by dot blotting experiments analyzing serial dilutions of purified TTFC, and binding assay supernatant. Filters were then visualized by the ECL-prime (Amersham Pharmacia Biotech) method and subjected to densitometric analysis by Quantity One 1-D Analysis Software (Bio-Rad).

### Flow cytometry

A total of 5.0 × 10^5^ TTFC-adsorbed spores were blocked with 1xPBS containing 3% of fetal bovine serum for 30 min at 25 ºC and subsequently incubated with anti-TTFC specific rabbit polyclonal antibodies diluted starting at 1:20 for 1 h at 25 ºC. After three washes with PBS, fluorescein isothiscyanate (FITC)-conjugated anti-rabbit IgG (1:50; Invitrogen) was added and incubated for 30 min at 25 ºC, followed three washes with PBS. To evaluate the non‐specific fluorescence, free spores stained or not with primary and secondary antibodies were analyzed. Samples were then resuspended in 400 µl of PBS and analyzed using by BD Accuri™ C6 Cytometer and BD Accuri™ C6 Software (BD Biosciences, Inc., Milan, Italy) collecting 100,000 events.

### Animals, probiotic supplementation and vaccination

Male C57BL/6 mice (Charles River, Italy) 8 weeks old were singularly caged in a temperature-controlled room (23  ±  1  °C) with a 12-h light/dark cycle (6.30 am–6.30 pm). Treatment, housing, and euthanasia of animals met the guidelines set by the Italian Health Ministry. All experimental procedures were approved by the “Comitato Etico-Scientifico per la Sperimentazione Animale” of the Federico II University of Naples (Italy). We used 40 mice that were divided in 6 groups named Naïve (n = 4), Probiotic (n = 4), TTFC (n = 8), TTFC+Probiotic (n = 8), Sp-TTFC (n = 8), and Sp-TTFC + Probiotic (n = 8). The Naïve, TTFC, and Sp-TTFC were fed with a commercial feed (Standard chow, Mucedola 4RF21, Italy), free of chemotherapeutic agents; whereas, the Probiotic, TTFC + Probiotic, and Sp-TTFC + Probiotic groups received the same commercial feed but supplemented with 1 × 10^6^ spores of *B. toyonensis* per gram of food from 7 days before the first vaccination for diet adaptation.

Mice were vaccinated by the intranasal route on day 0 and received a booster on days 14 and 28 of the experiment. TTFC and TTFC + Probiotic groups were vaccinated with 2.0 µg of purified TTFC suspended in 50 mM Sodium Citrate buffer. The Sp-TTFC and Sp-TTFC + Probiotic groups were vaccinated with 2.0 × 10^9^ spore-adsorbed with 2.0 µg of TTFC in a volume of 20 µl of 50 mM Sodium Citrate buffer. The naïve and probiotic groups were not vaccinated. Blood samples were collected by the submandibular puncture on days 0, 14, 21 and 35. After collection, serum was separated, labelled and stored − 20 °C until analysis. Fecal pellets were collected on day 0, 14, 21 and 35 to monitor the induction of the TTFC-specific IgA.

### Antibody analysis

Indirect ELISA was performed to evaluated serum levels of total IgG and IgG1, IgG2b, IgG2c, and IgG3 specific against TTFC. Microtitre plates (96 well, Corning, Lowell, MA, USA) were coated overnight at 4 °C with 0,2 µg of TTFC per well and subsequently washed with phosphate-buffered saline containing 0.05% Tween 20 (PBS-T). Plates were blocked with PBS containing 5% of Milk. Samples of individual serum samples were serially two-fold diluted starting at 1:2 to 20,480 and added to the plates in triplicate. After incubation at 37 °C for 1 h, the plates were washed with PBS-T, followed by addition of horseradish peroxidase (HRP)-conjugated rabbit anti-sheep IgG whole molecule antibodies (1:4000 dilution, Sigma-Aldrich, St. Louis, MO, USA). Following a further incubation at 37 °C 1 h, the plates were promptly washed again with PBS-T and added developing solution containing 10 ml of substrate buffer, 0.004 g of Ortho-Phenylenediamine (OPD) (Sigma-Aldrich) and 15 μl of H_2_O_2_ were added, and incubated in the dark at room temperature for 15 min and then stopped by adding 2 N sulphuric acid. Absorbance values were measured in a microplate reader (Thermo Fischer Scientific, Waltham, MA, USA) with a 492-nm filter. IgG isotype analysis performed according to the instruction manual of the Mouse Monoclonal Antibody Isotyping Reagents kit (Sigma-Aldrich), following the same protocol above describe. For ELISA analysis of fecal IgA, we followed the procedure described by [46], using approximately 0.1 g of fecal pellets that had been suspended in 1% of PBS and 1 mM of phenylmethylsulfonyl fluoride (Sigma-Aldrich), incubated at 4 ºC overnight, and stored at − 20 ºC prior to ELISA. The fecal extracts were tested by indirect ELISA for the presence of TTFC-specific IgA using a similar method to that shown above. Secretery IgAs were detected using Goat Anti-Mouse IgA alpha chain (HRP) (1:1000 dilution, Abcam, Cambridge, UK).

### Spleen cell cultures and cytokine production

Mice were sacrificed on day 35 and their spleen collected and macerated. Spleen cells (2.0 × 10^6^) were cultured in RPMI 1640 (Gibco, Grand Island, NY, USA) containing 10% fetal bovine serum (Gibco) and antibiotic and antifungal agents (penicillin 10,000 IU/ml, streptomycin 10 mg/ml and amphotericin B 25 mg/mL) (Gibco) in 24-well plates (Corning) and incubated for 24 h at 37 °C in 5% CO_2_ atmosphere. Culture medium was replaced after 24 h and the cells were stimulated with 10 µg of TTFC, 10 µg of concanavalin A (ConA; Sigma-Aldrich), and with RPMI 1640, and incubated for 72 h under the same conditions. ConA and RPMI were used as positive and negative control, respectively, for cell stimuli. Supernatants were harvested from cultures and analysed by Murine ELISA kit to detected production of followed cytokines IL-4 (Elabscience, USA), IL-6 (Diaclone, France), IL-10 (Diaclone), IL-12 (Elabscience), and IFN-γ (Diaclone). The assays were performed according to the manufacturers’ instructions.

### Microbiota identification by 16S rRNA sequencing

Total genomic DNA was extracted from 220 mg of mice fecal samples collected at the end of treatments (day 35) from all experimental groups using the QIAamp DNA Stool Mini Kit (QIAGEN) following the manufacturer’s instructions.

Partial 16S rRNA gene sequences were amplified from extracted DNA using primer pair Probio_Uni and Probio_Rev, which target the V3 region of the 16S rRNA gene sequence [47]. 16S rRNA gene amplification and amplicon checks were carried out as previously described [47]. 16S rRNA gene sequencing was performed using a MiSeq (Illumina) at the DNA sequencing facility of GenProbio srl (www.genprobio.com) according to the protocol previously reported [47].

Following sequencing and demultiplexing, the obtained reads of each sample were filtered to remove low quality and polyclonal sequences. All quality-approved, trimmed and filtered data were exported as.fastq files. The .fastq files were processed using a script based on the QIIME software suite [48]. Paired-end reads pairs were assembled to reconstruct the complete Probio_Uni/Probio_Rev amplicons. Quality control retained those sequences with a length between 140 and 400 bp and mean sequence quality score > 20. Sequences with homopolymers > 7 bp and mismatched primers were omitted.

In order to calculate downstream diversity measures (alpha and beta diversity indices, Unifrac analysis), 16S rRNA Operational Taxonomic Units (OTUs) were defined at ≥ 100% sequence homology using DADA2 and OTUs not encompassing at least 2 sequences of the same sample were removed. All reads were classified to the lowest possible taxonomic rank using QIIME2 [48, 49] and the SILVA database v. 132 as reference dataset [50]. Biodiversity of the samples (alpha-diversity) was calculated with Chao1 and Shannon indexes. Similarities between samples (beta-diversity) were calculated by weighted uniFrac [51]. The range of similarities is calculated between the values 0 and 1. PCoA representations of beta-diversity were performed using QIIME2 [48, 49].

### Statistical analysis

The data were analyzed using GraphPad Prism version 7 (USA). Differences among the various experimental groups were determined by the one-way ANOVA or two-way analysis of variance (ANOVA) followed by Tukey’s Multiple Comparisons test. The analysis of the fecal microbial composition was performed with SPSS software v. 25 (www.ibm.com/software/it/analytics/spss/). Analysis of Variance (ANOVA) was performed to compare differential abundance of bacterial genera. For multiple comparison, the post hoc analysis LSD (least significant difference) was calculated and differences with a p value < 0.05 were considered significant. The correlation test was performed by the Pearson method using the “cor.test” function from the “stats” R package [52].

## Supplementary information

**Additional file 1: Figure S1.** Dot plots of the cytofluorimeter analysis. The dot plots show the forward scatter (FSC-A) vs Fluorescence intensity distribution of free spores without antibodies (Sp), free (Sp (Ab^1^/Ab^2^)) and TTFC-adsorbed (Sp-TTFC (Ab^1^/Ab^2^)) spores incubated with polyclonal anti-TTFC and FITC-conjugated secondary antibodies. In all cases 100,000 spores were analyzed. Regions boxed in pink contain events (spores) with a fluorescence intensity higher than 1000 a.u. The percentage of positive events is reported for each graphs.

**Additional file 2: Figure S2.** Alpha diversity rarefaction plots. Estimation of the microbial taxa richness and diversity in fecal samples, based on Chao 1 (A) and Shannon (B) indexes. The number of observed OTUs in each sample is also reported (C).

**Additional file 3: Figure S3.** Fecal bacterial composition. Relative Operational Taxonomic Units (OTUs) abundance at the Family (A) and Genus (B) level in the six experimental groups, reported as mean values within each group. Only Taxa represented by OTUs abundance > 1% have been considered for the analysis.

## Data Availability

All data generated during this study are available from the corresponding author on reasonable request.

## References

[1] Zhang L, Wang W, Wang S (2015). Effect of vaccine administration modality on immunogenicity and efficacy. Expert Rev Vaccines..

[2] Nizard M, Diniz MO, Roussel H, Tran T, Ferreira LCS, Badoual C, Tartour E (2014). Novel strategies and applications for the control of pathogens and tumors at mucosal sites. Hum Vaccin Immunother.

[3] Kim S-H, Jang Y-S (2017). The development of mucosal vaccines for both mucosal and systemic immune induction and the roles played by adjuvants. Clin Exp Vaccine Res.

[4] Siegrist CA, Plotkin SA, Orenstein WA, Offit PA (2013). Vaccine immunology. Vaccines book.

[5] Woodrow KA, Bennett KM, Lo DD (2012). Mucosal vaccine design and delivery. Ann Rev Biomed Eng..

[6] Ricca E, Baccigalupi L, Cangiano G, De Felice M, Isticato R (2014). Mucosal vaccine delivery by non-recombinant spores of *Bacillus subtilis*. Microb Cell Fact.

[7] Ding C, Ma J, Dong Q, Liu Q (2018). Live bacterial vaccine vector and delivery strategies of heterologous antigen: a review. Immunol Lett.

[8] Hinc K, Stasiłojd M, Piątek I, Peszyoska-Sularz G, Isticato R, Ricca E, Obuchowski M, Iwanicki A (2014). Mucosal adjuvant activity of IL-2 presenting spores of *Bacillus subtilis* in a murine model of *Helicobacter pylori* vaccination. PLoS ONE.

[9] Fang Y, Polkb DB (2011). Probiotics and immune health. Curr Opin Gastroenterol..

[10] Beirão BCB, Ingberman M, Fávaro C, Mesa D, Bittencourt LC, Fascina VB, Caron LF (2018). Effect of an *Enterococcus faecium* probiotic on specific IgA following live *Salmonella Enteritidis* vaccination of layer chickens. Avian Pathol..

[11] Yeh TL, Shih PC, Liu SJ, Lin CH, Liu JM, Lei WT, Lin CY (2018). The influence of prebiotic or probiotic supplementation on antibody titers after influenza vaccination: a systematic review and meta-analysis of randomized controlled trials. Drug Des Devel Ther..

[12] Roos TB, de Moraes CM, Sturbelle RT, Dummer LA, Fischer G, Leite FPL (2018). Probiotics *Bacillus toyonensis* and *Saccharomyces boulardii* improve the vaccine immune response to *Bovine herpesvirus* type 5 in sheep. Res Vet Sci.

[13] Santos FDS, Menegon YA, Piraine REA, Rodrigues PRC, Cunha RC, Leite FPL (2018). *Bacillus toyonensis* improves immune response in the mice vaccinated with recombinant antigen of bovine herpesvirus type 5. Benef Microbes..

[14] Jiménez G, Urdiain M, Cifuentes A, López-López A, Blanch AR, Tamames J, Kämpfer P, Kolstø AB, Ramón D, Martínez JF, Codoñer FM, Rosselló-Móra R (2013). Description of *Bacillus toyonensis* sp. nov., a novel species of the *Bacillus cereus* group, and pairwise genome comparisons of the species of the group by means of ANI calculations. Syst Appl Microbiol..

[15] Kantas D, Papatsiros VG, Tassis PD, Giavasis I, Bouki P, Tzika ED (2013). A feed additive containing *Bacillus toyonensis* (Toyocerin) protects against enteric pathogens in postweaning piglets. J Appl Microbiol.

[16] Wells JM, Wilson PW, Norton PM, Gasson MJ, Le Page RW (1993). *Lactococcus lactis*: high-level expression of tetanus toxin fragment C and protection against lethal challenge. Mol Microbiol.

[17] Isticato R, Cangiano G, Tran TH, Ciabattini A, Medaglini D, Oggioni MR, De Felice M, Pozzi G, Ricca E (2001). Surface display of recombinant proteins on *Bacillus subtilis* spores. J Bacteriol.

[18] Duc LH, Huynh HA, Fairweather N, Ricca E, Cutting SM (2003). Bacterial spores as vaccine vehicles. Infect Immun.

[19] Mauriello EMF, Cangiano G, Maurano F, Saggese V, De Felice M, Rossi M, Ricca E (2007). Germination-independent induction of cellular immune response by *Bacillus subtilis* spores displaying the C fragment of the tetanus toxin. Vaccine..

[20] Huang JM, Hong HA, Van Tong H, Hoang TH, Brisson A, Cutting SM (2010). Mucosal delivery of antigens using adsorption to bacterial spores. Vaccine..

[21] Isticato R, Ricca E (2014). Spore surface display. Microbiol Spectr..

[22] Isticato R, Ricca E, Baccigalupi L. Spore adsorption as a nonrecombinant display system for enzymes and antigens. J Vis Exp. 2019; (145):e59102. 10.3791/59102. 10.3791/59102 30958471

[23] Mauriello EMF, Duc LH, Isticato R, Cangiano G, Hong HA, De Felice M, Ricca E, Cutting SM (2004). Display of heterologous antigens on the *Bacillus subtilis* spore coat using cotC as a fusion partner. Vaccine.

[24] Isticato R, Sirec T, Treppiccione L, Maurano F, De Felice M, Rossi M, Ricca E (2013). Non-recombinant display of the B subunit of the heat labile toxin of *Escherichia coli* on wild type and mutant spores of *Bacillus subtilis*. Microb Cell Fact.

[25] Hong HA, Hitri K, Hosseini S, Kotowicz N, Bryan D, Mawas F, Wilkinson AJ, van Broekhoven A, Kearsey J, Cutting SM (2017). Mucosal antibodies to the C terminus of toxin A prevent colonization of *Clostridium difficile*. Infect Immun..

[26] Valdez A, Yepiz G, Ricca E, Olmos-Soto J (2014). First *Litopenaeus vannamei* WSSV 100% oral vaccination protection using CotC::vp26 fusion protein displayed on *Bacillus subtilis* spores surface. J Appl Microbiol.

[27] Nguyen AT, Pham CK, Pham HT, Pham HL, Nguyen AH, Dang LT, Huynh HA, Cutting SM, Phan TN (2014). *Bacillus subtilis* spores expressing the VP28 antigen: a potential oral treatment to protect *Litopenaeus vannamei* against white spot syndrome. FEMS Microbiol Lett.

[28] Sibley L, Reljic R, Radford DS, Huang JM, Hong HA, Cranenburgh RM, Cutting SM (2014). Recombinant *Bacillus subtilis* spores expressing MPT64 evaluated as a vaccine against tuberculosis in the murine model. FEMS Microbiol Lett.

[29] Youngman P, Perkins JB, Losick R (1984). A novel method for the rapid cloning in *Escherichia coli* of *Bacillus subtilis* chromosomal DNA adjacent to Tn917 insertion. Mol Gen Genet.

[30] Donadio G, Lanzilli M, Sirec T, Ricca E, Isticato R (2016). Localization of a red fluorescence protein adsorbed on wild type and mutant spores of *Bacillus subtilis*. Microb Cell Fact.

[31] Sirec T, Strazzulli A, Isticato R, De Felice M, Moracci M, Ricca E (2012). Adsorption of beta-galactosidase of *Alicyclobacillus acidocaldarius* on wild type and mutants spores of *Bacillus subtilis*. Microb Cell Fact.

[32] Finkelman FD, Holmes J, Katona IM, Urban JF, Beckmam MP, Park LS, Schooleey KA, Coffman RL, Mosmann TR, Paul WE (1990). Lymphokine control of in vivo immunoglobulin isotype selection. Annu Rev Immunol.

[33] Germann T, Bongartz M, Dlugonska H, Hess H, Schmitt E, Kolbe L, Kölsch E, Podlaski FJ, Gately MK, Rüde E (1995). Interleukin-12 profoundly up-regulates the synthesis of antigen-specific complement-fixing IgG2a, IgG2b and IgG3 antibody subclasses in vivo. Eur J Immunol.

[34] Jones SA (2005). Directing transition from innate to acquired immunity: defining a role for IL-6. J Immunol..

[35] Hunter CA, Jones SA (2015). IL-6 as a keystone cytokine in health and disease. Nat Immunol.

[36] Roos TB, de Lara AP, Dummer LA, Fischer G, Leite FP (2012). The immune modulation of *Bacillus cereus* var. Toyoi in mice immunized with experimental inactivated *Bovine Herpesvirus* type 5 vaccine. Vaccine..

[37] Ouyang W, Rutz S, Crellin NK, Valdez PA, Hymowitz SG (2011). Regulation and functions of the IL-10 family of cytokines in inflammation and disease. Annu Rev Immunol.

[38] Moore BB, Moore TA, Toews GB (2001). Role of T- and B-lymphocytes in pulmonary host defences. Eur Respir J.

[39] Filipe-Santos O, Bustamante J, Chapgier A, Vogt G, de Beaucoudrey L, Feinberg J, Jouanguy E, Boisson-Dupuis S, Fieschi C, Picard C, Casanova JL (2006). Inborn errors of IL-12/23- and IFN-gamma-mediated immunity: molecular, cellular, and clinical features. Semin Immunol.

[40] Di Luccia B, D’Apuzzo E, Varriale F, Baccigalupi L, Ricca E, Pollice A (2016). *Bacillus megaterium* SF185 induces stress pathways and affects the cell cycle distribution of human intestinal epithelial cells. Benef Microbes..

[41] Li K, Zhang L, Xue J, Yang X, Dong X, Sha L, Lei H, Zhang X, Zhu L, Wang Z, Li X, Wang H, Liu P, Dong Y, He L (2019). Dietary inulin alleviates diverse stages of type 2 diabetes mellitus via anti-inflammation and modulating gut microbiota in db/db mice. Food Funct..

[42] Chung WSF, Walker AW, Louis P, Parkhill J, Vermeiren J, Bosscher D, Ducan SH, Flint HJ (2016). Modulation of the human gut microbiota by dietary fibres occurs at the species level. BMC Biol.

[43] Ichikawa-Seki M, Motooka D, Kinami A, Murakoshi F, Takahashi Y, Aita J, Hayashi K, Tashibu A, Nakamura S, Iida T, Horii T, Nishikawa Y (2019). Specific increase of *Fusobacterium* in the faecal microbiota of neonatal calves infected with *Cryptosporidium parvum*. Sci Rep..

[44] Martín R, Miquel S, Benevides L, Bridonneau C, Robert V, Hudault S, Chain F, Berteau O, Azevedo V, Chatel JM, Sokol H, Bermúdez-Humarán LG, Thomas M, Langella P (2017). Functional characterization of novel *Faecalibacterium prausnitzii* strains isolated from healthy volunteers: a step forward in the use of *F. prausnitzii* as a next-generation probiotic. Front. Microbiol..

[45] Cutting S, Vander Horn PB, Harwood C, Cutting S (1990). Genetic analysis. Molecular biological methods for *Bacillus*.

[46] Robinson K, Chamberlain LM, Schofield KM, Wells JM, Le Page RW (1997). Oral vaccination of mice against tetanus with recombinant *Lactococcus lactis*. Nat Biotechnol.

[47] Milani C, Hevia A, Foroni E, Duranti S, Turroni F, Lugli GA, Sanchez B, Martin R, Gueimonde M, van Sinderen D, Margolles A, Ventura M (2013). Assessing the fecal microbiota: an optimized ion torrent 16S rRNA gene-based analysis protocol. PLoS ONE.

[48] Caporaso JG, Kuczynski J, Stombaugh J, Bittinger K, Bushman FD, Costello EK, Fierer N, Gonzalez-Peña A, Goodrich JK, Gordon JI, Huttley GA, Kelley ST, Knights D, Koenig JE, Ley RE, Lozupone CA, McDonald D, Muegge BD, Pirrung M, Reeder J, Sevinsky JR, Turnbaugh PJ, Walters WA, Widmann J, Yatsunenko T, Zaneveld J, Knight R (2010). QIIME allows analysis of high-throughput community sequencing data. Nat Methods.

[49] Bokulich NA, Kaehler BD, Rideout JR, Dillon M, Bolyen E, Knight R, Huttley GA, Gregory Caporaso J (2018). Optimizing taxonomic classification of marker-gene amplicon sequences with QIIME 2′s q2-feature-classifier plugin. Microbiome..

[50] Quast C, Pruesse E, Yilmaz P, Gerken J, Schweer T, Yarza P, Peplies J, Glöckner FO (2013). The SILVA ribosomal RNA gene database project: improved data processing and web-based tools. Nucleic Acids Res.

[51] Lozupone C, Knight R (2005). UniFrac: a new phylogenetic method for comparing microbial communities. Appl Environ Microbiol.

[52] R CORE Team. R: a language and environment for statistical computing. R Foundation for Statistical Computing, Wien, Austria. ISBN 3-900051-07-0; 2013. URL http://www.R-project.org/.

