# Supplementary material for: A probiotic treatment increases the immune response induced by the nasal delivery of spore-adsorbed TTFC
Source: Microb Cell Fact. 2020 Feb 19;19:42. doi: 10.1186/s12934-020-01308-1 (PMC7029466; doi:10.1186/s12934-020-01308-1)
Supplement: Supplementary file 1 — Additional file 1: Figure S1. Dot plots of the cytofluorimeter analysis. The dot plots show the forward scatter (FSC-A) vs Fluorescence intensity distribution of free spores without antibodies (Sp), free (Sp (Ab1/Ab2)) and TTFC-adsorbed (Sp-TTFC (Ab1/Ab2)) spores incubated with polyclonal anti-TTFC and FITC-conjugated secondary antibodies. In all cases 100,000 spores were analyzed. Regions boxed in pink contain events (spores) with a fluorescence intensity higher than 1000 a.u. The percentage of positive events is reported for each graphs. [file 12934_2020_1308_MOESM1_ESM.pptx]

## Slide 1
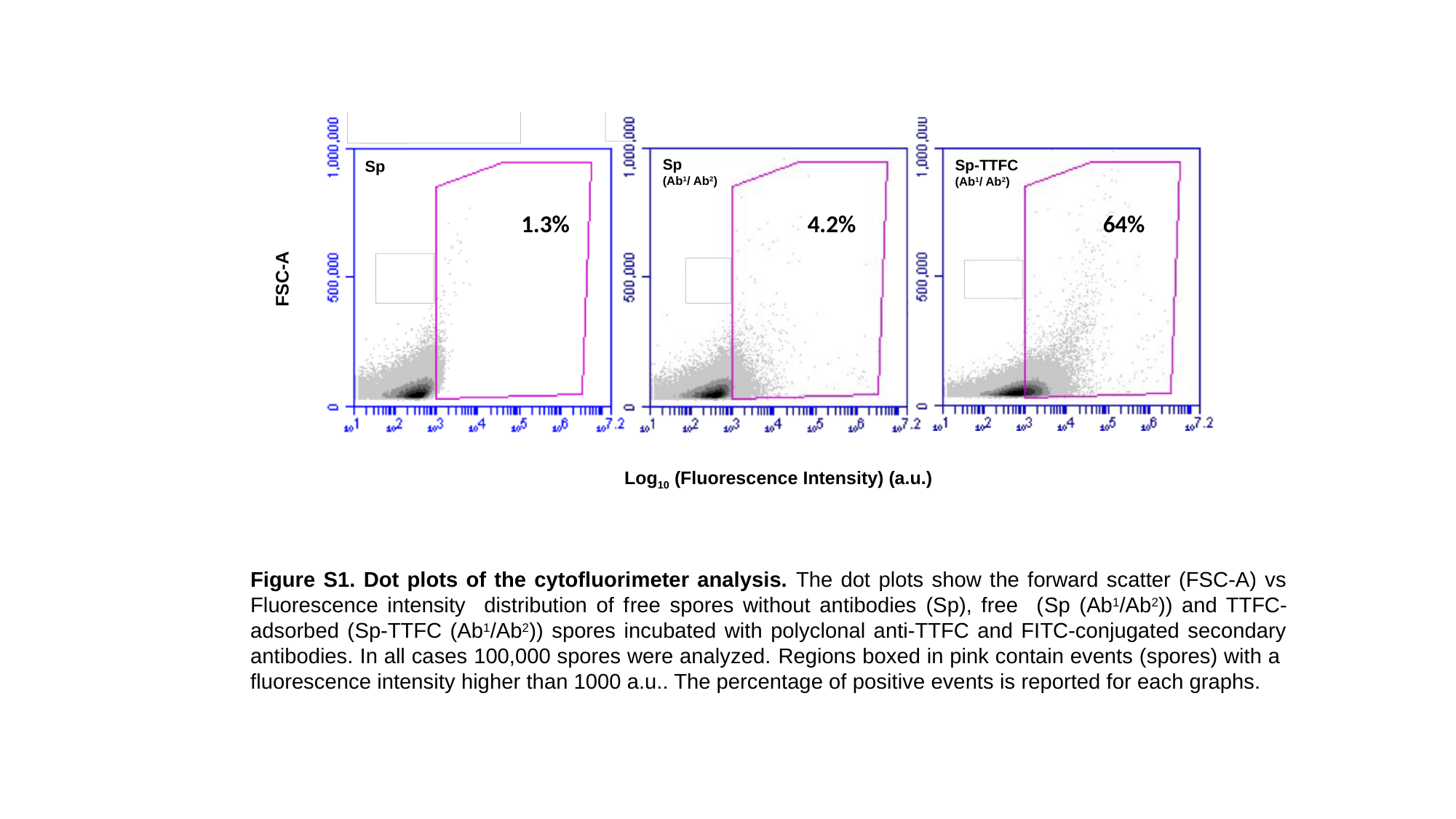

1.3%
4.2%
64%
FSC-A
 Log10 (Fluorescence Intensity) (a.u.)
Sp
(Ab1/ Ab2)
Sp-TTFC
(Ab1/ Ab2)
Sp
Figure S1. Dot plots of the cytofluorimeter analysis. The dot plots show the forward scatter (FSC-A) vs Fluorescence intensity distribution of free spores without antibodies (Sp), free (Sp (Ab1/Ab2)) and TTFC-adsorbed (Sp-TTFC (Ab1/Ab2)) spores incubated with polyclonal anti-TTFC and FITC-conjugated secondary antibodies. In all cases 100,000 spores were analyzed. Regions boxed in pink contain events (spores) with a fluorescence intensity higher than 1000 a.u.. The percentage of positive events is reported for each graphs.
