# Supplementary material for: A probiotic treatment increases the immune response induced by the nasal delivery of spore-adsorbed TTFC
Source: Microb Cell Fact. 2020 Feb 19;19:42. doi: 10.1186/s12934-020-01308-1 (PMC7029466; doi:10.1186/s12934-020-01308-1)
Supplement: Supplementary file 2 — Additional file 2: Figure S2. Alpha diversity rarefaction plots. Estimation of the microbial taxa richness and diversity in fecal samples, based on Chao 1 (A) and Shannon (B) indexes. The number of observed OTUs in each sample is also reported (C). [file 12934_2020_1308_MOESM2_ESM.pptx]

## Slide 1
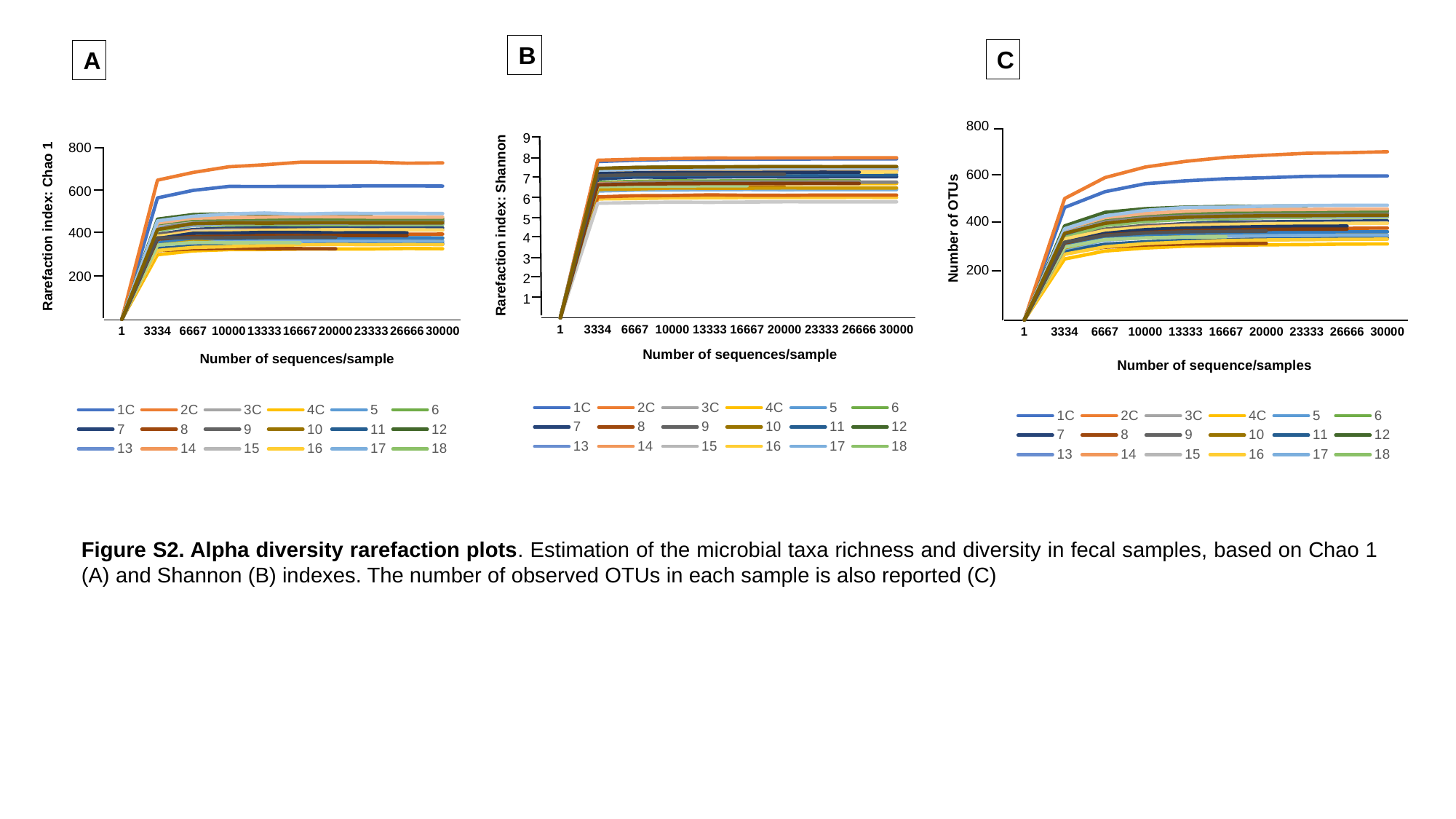

B
9
8
7
5
3
1
[unsupported chart]
6
4
2
Rarefaction index: Shannon
Number of sequences/sample
C
800
600
400
200
### Chart
| Category | 1C | 2C | 3C | 4C | 5 | 6 | 7 | 8 | 9 | 10 | 11 | 12 | 13 | 14 | 15 | 16 | 17 | 18 | 19 | 20 | 21 | 22 | 23 | 24 | 25 | 26 | 27 | 28 | 29 | 30 | 31 | 32 | 33 | 34 |
|---|---|---|---|---|---|---|---|---|---|---|---|---|---|---|---|---|---|---|---|---|---|---|---|---|---|---|---|---|---|---|---|---|---|---|
| 1 | 1.0 | 1.0 | 1.0 | 1.0 | 1.0 | 1.0 | 1.0 | 1.0 | 1.0 | 1.0 | 1.0 | 1.0 | 1.0 | 1.0 | 1.0 | 1.0 | 1.0 | 1.0 | 1.0 | 1.0 | 1.0 | 1.0 | 1.0 | 1.0 | 1.0 | 1.0 | 1.0 | 1.0 | 1.0 | 1.0 | 1.0 | 1.0 | 1.0 | 1.0 |
| 3334 | 482.8 | 520.9 | 286.7 | 262.2 | 307.8 | 319.2 | 333.4 | 288.0 | 353.6 | 370.4 | 296.9 | 403.9 | 378.7 | 334.1 | 376.9 | 282.5 | 306.5 | 357.2 | 369.0 | 325.3 | 372.4 | 310.1 | 328.3 | 390.5 | 376.7 | 390.3 | 330.6 | 348.0 | 391.8 | 312.9 | 333.3 | 330.6 | 333.8 | 372.3 |
| 6667 | 550.1 | 610.5 | 320.3 | 296.3 | 346.7 | 359.7 | 387.2 | 312.7 | 390.0 | 418.0 | 325.4 | 461.8 | 425.9 | 382.1 | 424.4 | 314.5 | 342.7 | 405.5 | 419.6 | 363.2 | 421.9 | 347.3 | 356.7 | 435.8 | 416.1 | 439.2 | 377.2 | 384.4 | 446.5 | 345.1 | 371.7 | 364.5 | 362.6 | 415.0 |
| 10000 | 584.4 | 655.7 | 334.2 | 309.1 | 357.1 | 374.1 | 403.4 | 322.2 | 405.9 | 433.5 | 335.7 | 477.4 | 444.8 | 399.5 | 443.3 | 327.9 | 353.0 | 422.6 | 438.5 | 375.3 | 440.6 | 362.6 | 366.2 | 453.8 | 432.1 | 456.7 | 395.2 | 396.0 | 470.3 | 352.3 | 387.8 | 375.6 | 373.3 | 432.5 |
| 13333 | 596.7 | 680.0 | 340.4 | 317.2 | 362.7 | 381.5 | 413.8 | 326.7 | 410.8 | 446.4 | 342.2 | 484.3 | 451.9 | 406.3 | 454.9 | 335.0 | 358.9 | 432.0 | 448.8 | 383.1 | 447.0 | 371.2 | 371.5 | 461.3 | 439.3 | 466.7 | 401.5 | 405.7 | 482.7 | 355.8 | 394.5 | 383.0 | 378.3 | 440.8 |
| 16667 | 605.7 | 697.1 | 345.2 | 320.6 | 363.7 | 388.6 | 419.6 | 328.2 | 414.4 | 450.1 | 345.0 | 487.0 | 457.6 | 409.6 | 458.4 | 341.3 | 360.7 | 434.3 | 452.8 | 386.2 | 453.2 | 373.6 | 374.6 | 464.4 | 441.4 | 472.1 | 408.8 | 408.9 | 483.8 | 356.9 | 398.0 | 386.0 | 380.4 | 444.5 |
| 20000 | 610.1 | 706.2 | 348.7 | 322.9 | 364.6 | 390.5 | 421.0 | 329.0 | 416.3 | 451.8 | 346.8 | 488.5 | 459.4 | 411.6 | 461.5 | 343.7 | 362.4 | 438.9 | 456.0 | 389.0 | 455.1 | 376.8 | 376.4 | 466.7 | 444.3 | 473.0 | 412.7 | 412.0 | 488.8 | None | 400.9 | 389.3 | 381.6 | 447.4 |
| 23333 | 615.7 | 714.5 | 349.5 | 323.8 | None | 391.9 | 423.3 | None | 416.8 | 453.8 | 348.6 | 489.0 | 460.9 | 412.2 | 463.7 | 344.6 | 363.2 | 440.8 | 457.0 | 391.4 | 456.2 | 378.3 | 378.2 | 467.1 | 446.1 | 474.3 | 413.4 | 413.3 | 490.8 | None | 402.8 | 389.8 | None | 447.6 |
| 26666 | 617.4 | 716.9 | 352.7 | 326.3 | None | 392.0 | 424.2 | None | 417.0 | 455.2 | 350.5 | None | 461.0 | 413.0 | 464.3 | 346.4 | 364.1 | 441.4 | 457.9 | 393.3 | 457.4 | 379.0 | 378.7 | 467.7 | 446.6 | 475.7 | 414.4 | 414.5 | 492.2 | None | 403.0 | 390.0 | None | 448.9 |
| 30000 | 617.7 | 721.2 | 354.7 | 326.4 | None | None | 425.4 | None | None | 455.7 | 350.7 | None | None | None | 464.9 | 347.0 | 364.3 | 441.6 | 458.0 | 394.6 | 458.4 | 379.0 | 378.9 | 468.0 | 446.8 | 476.0 | 415.7 | 415.0 | 492.3 | None | None | None | None | 449.0 |Number of OTUs
Number of sequence/samples
A
800
600
400
200
[unsupported chart]
Rarefaction index: Chao 1
Number of sequences/sample
Figure S2. Alpha diversity rarefaction plots. Estimation of the microbial taxa richness and diversity in fecal samples, based on Chao 1 (A) and Shannon (B) indexes. The number of observed OTUs in each sample is also reported (C)
