# Supplementary material for: A probiotic treatment increases the immune response induced by the nasal delivery of spore-adsorbed TTFC
Source: Microb Cell Fact. 2020 Feb 19;19:42. doi: 10.1186/s12934-020-01308-1 (PMC7029466; doi:10.1186/s12934-020-01308-1)
Supplement: Supplementary file 3 — Additional file 3: Figure S3. Fecal bacterial composition. Relative Operational Taxonomic Units (OTUs) abundance at the Family (A) and Genus (B) level in the six experimental groups, reported as mean values within each group. Only Taxa represented by OTUs abundance > 1% have been considered for the analysis. [file 12934_2020_1308_MOESM3_ESM.pptx]

## Slide 1
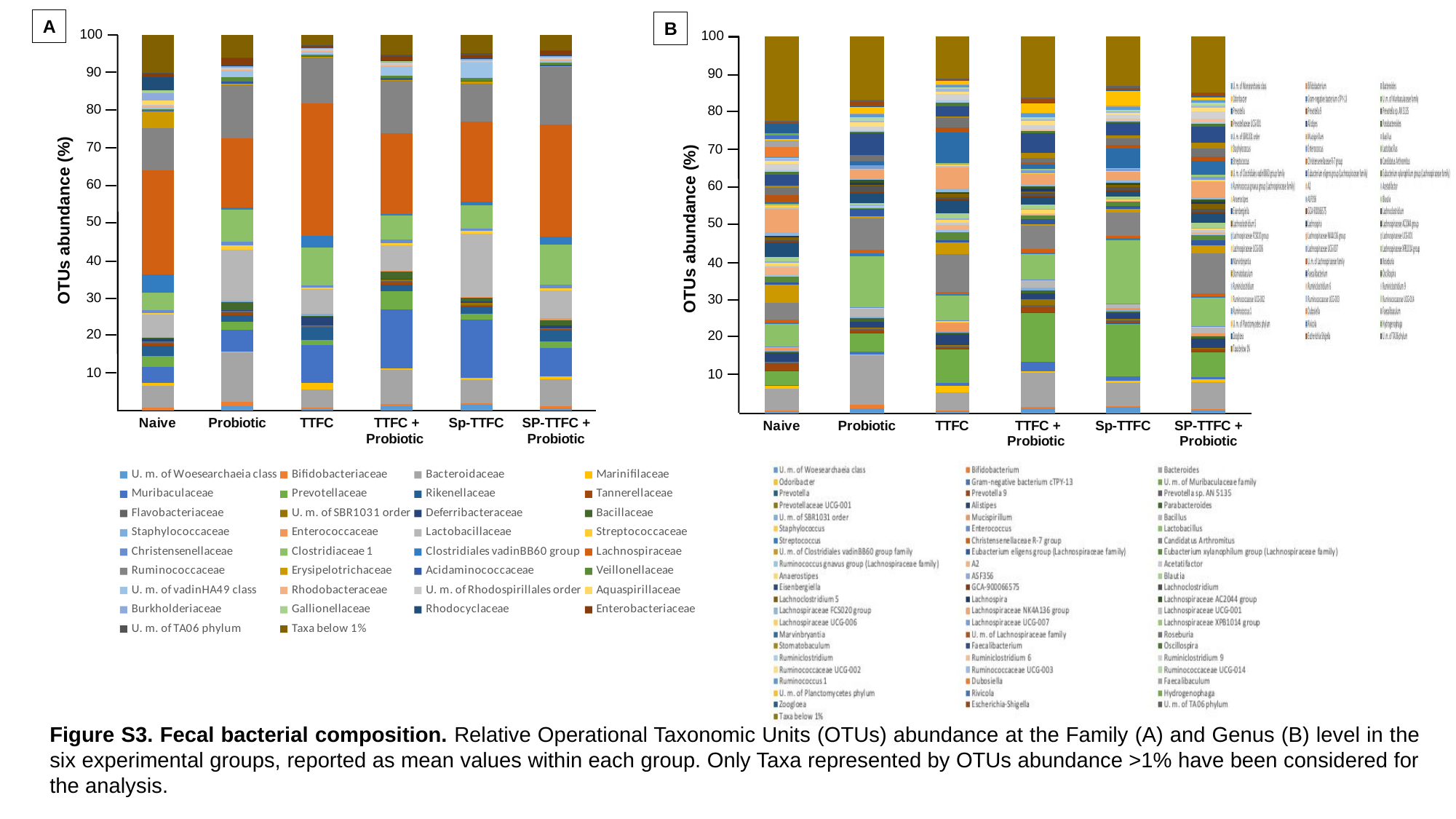

A
B
### Chart
| Category | U. m. of Woesearchaeia class | Bifidobacterium | Bacteroides | Odoribacter | Gram-negative bacterium cTPY-13 | U. m. of Muribaculaceae family | Prevotella | Prevotella 9 | Prevotella sp. AN 5135 | Prevotellaceae UCG-001 | Alistipes | Parabacteroides | U. m. of SBR1031 order | Mucispirillum | Bacillus | Staphylococcus | Enterococcus | Lactobacillus | Streptococcus | Christensenellaceae R-7 group | Candidatus Arthromitus | U. m. of Clostridiales vadinBB60 group family | Eubacterium eligens group (Lachnospiraceae family) | Eubacterium xylanophilum group (Lachnospiraceae family) | Ruminococcus gnavus group (Lachnospiraceae family) | A2 | Acetatifactor | Anaerostipes | ASF356 | Blautia | Eisenbergiella | GCA-900066575 | Lachnoclostridium | Lachnoclostridium 5 | Lachnospira | Lachnospiraceae AC2044 group | Lachnospiraceae FCS020 group | Lachnospiraceae NK4A136 group | Lachnospiraceae UCG-001 | Lachnospiraceae UCG-006 | Lachnospiraceae UCG-007 | Lachnospiraceae XPB1014 group | Marvinbryantia | U. m. of Lachnospiraceae family | Roseburia | Stomatobaculum | Faecalibacterium | Oscillospira | Ruminiclostridium | Ruminiclostridium 6 | Ruminiclostridium 9 | Ruminococcaceae UCG-002 | Ruminococcaceae UCG-003 | Ruminococcaceae UCG-014 | Ruminococcus 1 | Dubosiella | Faecalibaculum | U. m. of Planctomycetes phylum | Rivicola | Hydrogenophaga | Zoogloea | Escherichia-Shigella | U. m. of TA06 phylum | Taxa below 1% |
|---|---|---|---|---|---|---|---|---|---|---|---|---|---|---|---|---|---|---|---|---|---|---|---|---|---|---|---|---|---|---|---|---|---|---|---|---|---|---|---|---|---|---|---|---|---|---|---|---|---|---|---|---|---|---|---|---|---|---|---|---|---|---|---|---|
| Naive | 0.003518532899716999 | 0.003158823683372147 | 0.05808061881633258 | 0.008587166379582359 | 0.0025303339044218586 | 0.03737569660073187 | 0.00027093344238830384 | 0.02059238638822558 | 0.0013775837708227948 | 0.0013480211946099235 | 0.023149909779994406 | 0.0053908616197771925 | 0.00108238499357685 | 0.00765757579502958 | 0.002208758098197197 | 0.0005065050899948514 | 0.00018413041343415495 | 0.060716230850795354 | 0.0029641095068702584 | 0.007848305742149486 | 0.0457847885131728 | 0.04683159271735569 | 0.005902504106170084 | 0.016182993133000662 | 0.005974754602004365 | 0.01828085824904667 | 0.005267762032957703 | 0.006770812245416742 | 0.004743278577583324 | 0.011773954945674436 | 0.035295606305382476 | 0.00343740175130683 | 0.005297480414278321 | 0.0075305865792959885 | 0.004214405154270339 | 0.0 | 0.00878151138144707 | 0.06280168579311136 | 0.0036176972322991456 | 0.006058465467291446 | 0.0025065664458395216 | 0.001180906936527253 | 0.003727398480838953 | 0.0187172382130293 | 0.020392803658098875 | 0.004008651906237252 | 0.029372749340755584 | 0.008217360984834316 | 0.0044895614297595105 | 0.001606802189111706 | 0.014943615326522896 | 0.008852942775098339 | 0.0052809061398372895 | 0.0023395635170505727 | 0.0033905145491818227 | 0.0240465559468044 | 0.01805771931416121 | 0.003949985994215599 | 0.010357011656243413 | 0.005315613547211277 | 0.024600621611995856 | 0.0064874963283314034 | 0.0005893275161191779 | 0.2244710780211054 |
| Probiotic | 0.013163120264315603 | 0.010650514359528856 | 0.13040059463312584 | 0.0018991265345420648 | 0.008425036661366871 | 0.04953528419760141 | 0.0004005710651882704 | 0.009223725200969176 | 0.0007017437487158689 | 0.003366700761391092 | 0.016086420561795208 | 0.00872886282490666 | 0.0019577200246605787 | 0.0015574908450261336 | 0.022203249425291127 | 0.0012340081541563329 | 0.0021856632056821506 | 0.13546423014947137 | 0.007937988931015827 | 0.009558949011829831 | 0.08336342721468623 | 0.005311932233520571 | 0.0198531106631723 | 0.001972990699143096 | 0.0029064932793258997 | 0.0017896336555914701 | 0.001174462512492939 | 0.0 | 0.001072162679543835 | 0.006002571609056511 | 0.024615688917644925 | 0.0053673258752737416 | 0.015971445391344086 | 0.002365362587729467 | 0.006259544398451134 | 0.006431985989228079 | 0.003139236652541975 | 0.02627002713949609 | 0.00034265309298614016 | 0.0 | 0.010246480634137313 | 0.0 | 0.010517914833192244 | 0.0009773560989361318 | 0.015753455899509203 | 0.0 | 0.05746252123622073 | 0.00453125914458267 | 0.0009079308813556672 | 0.0026025789781091175 | 0.00857695422257187 | 0.011957773632671819 | 0.004594584550548139 | 0.008154680205031177 | 0.0097994185341457 | 0.0 | 0.00033669784713621 | 0.018760566388688477 | 0.0 | 0.0 | 0.0003236420044778974 | 0.01495794364140176 | 0.0035628095361254462 | 0.16708437657934969 |
| TTFC | 0.004950527248199215 | 0.003020076567112323 | 0.04862367083898741 | 0.017512606046159493 | 0.007206058758524009 | 0.09035842102336039 | 0.00016792105754990044 | 0.005564742630624306 | 0.00041739415709393825 | 0.0035689559623066484 | 0.029115767376799422 | 0.0041495545726574126 | 0.003100538475607947 | 0.022720141094043387 | 0.0011364666771633768 | 0.005838512672529514 | 2.1596779488242712e-05 | 0.06580392568883267 | 0.0037116514752859823 | 0.004949825242410479 | 0.1005150695509011 | 0.030752874269134495 | 0.007119330673319681 | 0.02022057780702258 | 0.005284368473750869 | 0.015033791325274708 | 0.005645603152003847 | 0.006809704020395094 | 0.004492731158351559 | 0.012655215647284319 | 0.0347182556284046 | 0.004377030846233595 | 0.004113205776866822 | 0.009119812702056381 | 0.0027339718608539233 | 0.0017018765629148412 | 0.010062616935441885 | 0.055489236937172635 | 0.00392078905303104 | 0.003421901015501609 | 0.004612788865954815 | 7.084912678451239e-05 | 0.0806010711645911 | 0.013741736821572549 | 0.02493104898633836 | 0.00479031119029236 | 0.026239854736080523 | 0.009387926371789814 | 0.006343953487709049 | 0.0023815707127305356 | 0.015502750531826847 | 0.005804129367944243 | 0.007334082014766673 | 0.0036755710805233236 | 0.00724222719536555 | 2.287701317715959e-05 | 0.0006349925620314169 | 0.009479990166606741 | 0.0 | 0.0 | 0.0 | 0.0040841374678787555 | 0.0011723505408603697 | 0.1118194628545237 |
| TTFC + Probiotic | 0.012873342334541177 | 0.004458010704229042 | 0.09114691567598109 | 0.0026000320648886235 | 0.026777829450168242 | 0.12895397305201145 | 0.0019425462560065721 | 0.015651613287288615 | 0.0038916421033186135 | 0.014681673387549613 | 0.01614192519551596 | 0.007774220480645961 | 0.005623773788138491 | 0.0005154361751364157 | 0.02069460439650561 | 0.0007132315797303261 | 0.0007580950387468468 | 0.06755990168642527 | 0.004474750076443727 | 0.009107246307526582 | 0.06171456234606879 | 0.004269689103622391 | 0.013525143785555098 | 0.00876763815815481 | 0.003070995518430952 | 0.002422887017836107 | 0.0016102844678452988 | 0.009570649189007923 | 0.0016716895446289713 | 0.01149537249887445 | 0.019125834001241078 | 0.003321888407620375 | 0.008045064157782915 | 0.0048828132371604325 | 0.006905863984082674 | 0.006083422215755745 | 0.003252124831206866 | 0.03011736625057465 | 0.00043703035767150985 | 0.0006198699521160862 | 0.00827393404346287 | 0.002998766171080479 | 0.012085415303509404 | 0.005260807594479853 | 0.012785887268356543 | 0.012977923781317542 | 0.051930970910550746 | 0.005209929436518177 | 0.00174838647414302 | 0.0020688789851027625 | 0.01266360989233992 | 0.010732758270764147 | 0.0044982850106850625 | 0.006305362275448941 | 0.011575626261119978 | 0.0 | 8.224690075655918e-05 | 0.02697976493847293 | 0.0 | 0.0 | 9.790971892171258e-05 | 0.009297333999564015 | 0.003128328496400767 | 0.16204692220096925 |
| Sp-TTFC | 0.017152446250256116 | 0.0026647896117967082 | 0.061762456702202075 | 0.005848024470977467 | 0.010345952872844956 | 0.1412465731939006 | 0.00025770709813201876 | 0.008014825872588243 | 0.00022014256561832227 | 0.0027326683238195755 | 0.016002070616318313 | 0.00379149760019291 | 0.006030448643688872 | 0.0030137388684261476 | 0.010028940225952947 | 0.0015791556124247351 | 0.0004260607136516954 | 0.16875290811992752 | 0.0054071319273357715 | 0.00603089434621397 | 0.06093882916680486 | 0.009514554705344399 | 0.009455997119050952 | 0.010199135128000653 | 0.0021048045237779195 | 0.0011712166564471487 | 0.0009126132059398644 | 0.0013646430529270718 | 0.0019250818601852999 | 0.005954787521805367 | 0.014277715623407005 | 0.0037148081142339444 | 0.0072356316660481205 | 0.005502980704991883 | 0.0035662140367473416 | 0.004012741033687977 | 0.003987306087632221 | 0.02373753972291531 | 0.0015718257503426205 | 0.0006289467677715503 | 0.005961095410124536 | 0.0008166163678324162 | 0.052938274410036276 | 0.009774973893638447 | 0.017909764440970477 | 0.006986469006225738 | 0.03336133745140089 | 0.003928932813468457 | 0.0018703083245381529 | 0.00555717723946695 | 0.009564599057036757 | 0.005007389613123472 | 0.005309554486791719 | 0.002838766343066483 | 0.007459145842721358 | 0.0 | 0.004315838940517424 | 0.041371900712661336 | 0.0 | 0.0 | 0.00020916532878831174 | 0.005399472618988021 | 0.005135136099973016 | 0.13119827551429925 |
| SP-TTFC + Probiotic | 0.0067139125059471364 | 0.0047258737181791795 | 0.07231511972961116 | 0.007361468690645638 | 0.00491475241396619 | 0.06803550904444425 | 0.0001408078751589466 | 0.006521717839636009 | 0.0001056510641175178 | 0.003437347920226873 | 0.02513867024157279 | 0.004905879524742625 | 0.0018548599936613153 | 0.006498562695244429 | 0.015545211128656158 | 0.0005802090556711355 | 0.002621434697382002 | 0.07512554943467162 | 0.0035276808227608157 | 0.009401825818515602 | 0.10600906329343944 | 0.020991464162063548 | 0.012689661394298349 | 0.01422366408653498 | 0.0044352203768630175 | 0.003051365566524606 | 0.005584082121134377 | 0.004161365993821995 | 0.0019855422493936592 | 0.014148127994482897 | 0.024317060593040774 | 0.004253500958438164 | 0.007577160271923621 | 0.01276103907195715 | 0.005429366396124462 | 0.00723586541099886 | 0.005053125101205124 | 0.04044160374625468 | 0.0030686311675549734 | 0.0028872607522550347 | 0.0077142380266191635 | 0.006138336395672887 | 0.036514800792071325 | 0.011071086189033661 | 0.021010697278567598 | 0.016648297620464665 | 0.04221194462363686 | 0.008282390222897477 | 0.0034652318977828602 | 0.00851373603199867 | 0.020369725912837942 | 0.00924388401546215 | 0.006351245347069934 | 0.00666645183459335 | 0.009060456945021932 | 0.0 | 0.00020030687834926146 | 0.007731160686039628 | 0.00016940698144993555 | 0.0 | 0.0005277371712990479 | 0.00866141364846599 | 0.0013109609706407775 | 0.1483602756069038 |100
90
80
70
60
50
OTUs abundance (%)
40
30
20
10
100
90
80
70
60
OTUs abundance (%)
50
40
30
20
10
### Chart
| Category | U. m. of Woesearchaeia class | Bifidobacteriaceae | Bacteroidaceae | Marinifilaceae | Muribaculaceae | Prevotellaceae | Rikenellaceae | Tannerellaceae | Flavobacteriaceae | U. m. of SBR1031 order | Deferribacteraceae | Bacillaceae | Staphylococcaceae | Enterococcaceae | Lactobacillaceae | Streptococcaceae | Christensenellaceae | Clostridiaceae 1 | Clostridiales vadinBB60 group | Lachnospiraceae | Ruminococcaceae | Erysipelotrichaceae | Acidaminococcaceae | Veillonellaceae | U. m. of vadinHA49 class | Rhodobacteraceae | U. m. of Rhodospirillales order | Aquaspirillaceae | Burkholderiaceae | Gallionellaceae | Rhodocyclaceae | Enterobacteriaceae | U. m. of TA06 phylum | Taxa below 1% |
|---|---|---|---|---|---|---|---|---|---|---|---|---|---|---|---|---|---|---|---|---|---|---|---|---|---|---|---|---|---|---|---|---|---|---|
| Naive | 0.0035206482202302103 | 0.00418916542172955 | 0.05812497724803923 | 0.009308403773765652 | 0.041024319655353456 | 0.0307570364040901 | 0.02645295320893271 | 0.005395019370961112 | 0.005429280066889473 | 0.0010831398183991279 | 0.007662237307108204 | 0.002210320187143898 | 0.0005068958948957292 | 0.00018416206261510129 | 0.06076742183123127 | 0.0032273198606127575 | 0.007951330176184856 | 0.047165539634402225 | 0.0468599677351483 | 0.2775590959807259 | 0.11268113794947114 | 0.04430613195263914 | 0.002457118545037387 | 0.003853274362021905 | 0.004007691301903174 | 0.0057264363188229105 | 0.001962904986611346 | 0.010365502915201856 | 0.020029496629193396 | 0.007594675776948053 | 0.0341227573739154 | 0.01162213955925041 | 0.0005896531158782554 | 0.10130184535464665 |
| Probiotic | 0.013174430608570606 | 0.011180103246877862 | 0.1305522364698729 | 0.002208302168960396 | 0.058353277912516696 | 0.022081990707066138 | 0.017085189898689415 | 0.008738571884494312 | 0.00033323948857266067 | 0.00195951359470523 | 0.0015584851702207744 | 0.02237632967083047 | 0.001235258910349968 | 0.00218728446736769 | 0.13558945892175608 | 0.010830253220515403 | 0.009673447055922185 | 0.0864405611891828 | 0.005316635853703784 | 0.18331356750577743 | 0.1416610230735965 | 0.003487042877406487 | 0.006023873216434933 | 0.01207990218479775 | 0.018775664820284418 | 0.0003148309459449857 | 0.005882251210052531 | 0.0 | 0.005422951908770597 | 0.0 | 0.0003239380320039018 | 0.018215568826310324 | 0.00356573691970261 | 0.060059078038742136 |
| TTFC | 0.004957762321718379 | 0.0032984100419682287 | 0.04869756697564014 | 0.017843589487535356 | 0.09952274788670969 | 0.013596552337289432 | 0.032608152506679484 | 0.004156008705010594 | 0.00020163004466873836 | 0.003104435094006491 | 0.022790888942445627 | 0.0011376745101836503 | 0.005844939057404343 | 2.161134163208852e-05 | 0.06589053811140082 | 0.00473761539289178 | 0.0050043660858293615 | 0.1011881979201275 | 0.03082063838705985 | 0.3519606279985323 | 0.12177562508276851 | 0.00201503901113109 | 0.0025772805836019575 | 0.00411716762367749 | 0.009494038451452221 | 0.0003370712057525125 | 0.00407928979557753 | 0.0 | 0.00283056169156124 | 0.0 | 4.114549045424622e-05 | 0.006254301247856323 | 0.0011740691376352019 | 0.0279204575297979 |
| TTFC + Probiotic | 0.012893380017253375 | 0.005029926874735688 | 0.09131756721576738 | 0.0034819463020984696 | 0.15697259568198427 | 0.04848183279714454 | 0.01810840926734151 | 0.0077897443286129835 | 0.00023848139959597685 | 0.005632104500077924 | 0.0005162553155718955 | 0.020901337680223182 | 0.000800074027138258 | 0.0007604586782297885 | 0.06769113209473133 | 0.005878944232117899 | 0.009325161103956437 | 0.06266267237890427 | 0.004276658008764307 | 0.21496663033636887 | 0.13973566902688317 | 0.002558587745604926 | 0.004289837600255387 | 0.007443885235518346 | 0.027021672364558495 | 0.0001855645798303933 | 0.006072834628594619 | 0.0 | 0.0053739101341945756 | 7.332839701381856e-05 | 0.00016878334671715083 | 0.013240376011821032 | 0.003133103553567417 | 0.05297713513482224 |
| Sp-TTFC | 0.017179272667926602 | 0.0027813040205682503 | 0.061860701550447934 | 0.006695346218522064 | 0.15386913185029982 | 0.016634016966706324 | 0.01823512294119143 | 0.003797541557236113 | 0.00015867250544768883 | 0.006040384321300539 | 0.0030185085630802066 | 0.01040621607202232 | 0.001581622585701869 | 0.00042689434364994664 | 0.1688865375653878 | 0.007103223489511224 | 0.006083127942457754 | 0.061957056022639644 | 0.009529649763981643 | 0.2126732950399246 | 0.10016821830506395 | 0.005863951329856843 | 0.0032294651392404837 | 0.006883945042609707 | 0.041438534173858406 | 0.0004966766445600499 | 0.004479185896596123 | 0.0 | 0.005298706150096101 | 0.0 | 0.00033315755980938775 | 0.008018572771964012 | 0.005143323031776078 | 0.04972863796656508 |
| SP-TTFC + Probiotic | 0.006723529667942805 | 0.005073050292022667 | 0.0724078236175381 | 0.008076155051547575 | 0.0744813870144397 | 0.018684409290316114 | 0.027800015210845994 | 0.004912379033058102 | 0.00034541310726877024 | 0.0018572562805321308 | 0.006510893396057143 | 0.015652031023548597 | 0.0005809704590476999 | 0.0026254714956493925 | 0.07522825856958802 | 0.004612656498647639 | 0.009591214530320444 | 0.10680021491266374 | 0.021031826129979805 | 0.2975491269919166 | 0.15283982173882046 | 0.0018933211301710062 | 0.004527692978849296 | 0.005607407312088019 | 0.0077431617500409 | 0.0008502139158102065 | 0.00588754562575807 | 0.00016961370478734683 | 0.004471417864787339 | 3.927776053920509e-05 | 0.0007733902103946651 | 0.012036152803901157 | 0.0013130165083508544 | 0.04130388412277064 |
Figure S3. Fecal bacterial composition. Relative Operational Taxonomic Units (OTUs) abundance at the Family (A) and Genus (B) level in the six experimental groups, reported as mean values within each group. Only Taxa represented by OTUs abundance >1% have been considered for the analysis.
